# Single Cell Analysis of Cultivated Fibroblasts from Chronic Pancreatitis and Pancreatic Cancer Patients

**DOI:** 10.3390/cells11162583

**Published:** 2022-08-19

**Authors:** Yoshiaki Sunami, Yijun Chen, Bogusz Trojanowicz, Matthias Sommerer, Monika Hämmerle, Roland Eils, Jörg Kleeff

**Affiliations:** 1 Department of Visceral, Vascular and Endocrine Surgery, Martin-Luther-University Halle-Wittenberg, University Medical Center Halle, 06120 Halle, Germany; 2 Institute of Pathology, Section for Experimental Pathology, Martin-Luther-University Halle-Wittenberg, University Medical Center Halle, 06120 Halle, Germany; 3 Center for Digital Health, Berlin Institute of Health (BIH) and Charité-Universitätsmedizin Berlin, 10117 Berlin, Germany; 4 Health Data Science Unit, Heidelberg University Hospital and BioQuant, 69120 Heidelberg, Germany

**Keywords:** cancer-associated fibroblasts, pancreatic cancer, chronic pancreatitis, single-cell RNA sequencing, cellular plasticity

## Abstract

Cancer-associated fibroblasts (CAFs) play a major role in the progression and drug resistance of pancreatic cancer. Recent studies suggest that CAFs exhibit functional heterogeneity and distinct transcriptomic signatures in pancreatic cancer. Pancreatic fibroblasts also form an integral component in pancreatic diseases such as chronic pancreatitis named disease-associated fibroblasts (DAFs). However, intra-tumoral heterogeneity of CAFs in pancreatic cancer patients and their pivotal role in cancer-related mechanisms have not been fully elucidated. Further, it has not been elucidated whether CAF subtypes identified in pancreatic cancer also exist in chronic pancreatitis. In this study, we used primary isolated fibroblasts from pancreatic cancer and chronic pancreatitis patients using the outgrowth method. Single-cell RNA sequencing (scRNA-seq) was performed, and bioinformatics analysis identified highly variable genes, including factors associated with overall survival of pancreatic cancer patients. The majority of highly variable genes are involved in the cell cycle. Instead of previously classified myofibroblastic (myCAFs), inflammatory (iCAFs), and antigen-presenting (ap) CAFs, we identified a myCAFs-like subtype in all cases. Most interestingly, after cell cycle regression, we observed 135 highly variable genes commonly identified in chronic pancreatitis and pancreatic cancer patients. This study is the first to conduct scRNAseq and bioinformatics analyses to compare CAFs/DAFs from both chronic pancreatitis and pancreatic cancer patients. Further studies are required to select and identify stromal factors in DAFs from chronic pancreatitis cases, which are commonly expressed also in CAFs potentially contributing to pancreatic cancer development.

## 1. Introduction

Pancreatic cancer is currently the fourth leading cause of cancer deaths and is projected to become the second most common cancer death in the United States by 2030 [1,2]. The dense stromal tumor microenvironment, which accounts for up to ~90% of the pancreatic tumor mass, is a major cause of resistance to chemotherapy and radiotherapy [3,4,5]. The major stromal compartment consists of CAFs, which are a source of extracellular matrix proteins and potential therapeutic targets [6]. Fibrotic stroma and generally termed disease-associated fibroblasts (DAFs) form an integral component in pancreatic diseases including pancreatic cancer (CAFs) and chronic pancreatitis [7]. CAF depletion can also have tumor-promoting effects, suggesting that CAFs exhibit distinct functional heterogeneity [5]. CAFs, which are derived from several cell types such as pancreatic stellate cells or mesenchymal stem cells, play a major role in the progression and drug resistance of pancreatic cancer [8,9]. Recent studies suggest that CAF subtypes exhibit distinct transcriptomic signatures in pancreatic cancer. Previous human and murine studies described several CAF subtypes such as myofibroblastic CAFs (myCAFs) expressing *Acta2*, (coding α-smooth muscle actin), *Vim* (vimentin), and *Ctgf* (connective tissue growth factor), *Col1a1*, *Col5a1*, and *Col6a1*, inflammatory CAFs (iCAFs) expressing *Il1*, *Il6*, *Il11*, and *Lif* (Leukemia inhibitory factor) [10], as well as antigen-presenting CAFs (apCAFs) expressing *Slpi*, *Saa3*, *Cd74*, *H2-Ab1*, *Nkain4* (Sodium/Potassium Transporting ATPase Interacting 4), and *Irf5* [11]. MyCAFs are associated with an extracellular matrix signature, while iCAFs are suggested to establish an immunosuppressive microenvironment [9]. ApCAFs may play an immunomodulatory role in pancreatic cancer [9]. In the current study, we used CAFs/DAFs isolated from pancreatic cancer and chronic pancreatitis patients via the outgrowth method to evaluate the presence of myCAF, iCAF, or apCAF subtypes in these specimens, and for further stratification of fibroblast subtypes associated with pancreatic disease development and progression.

## 2. Materials and Methods

### 2.1. Human Subjects

Human pancreas samples from patients with pancreatic cancer or chronic pancreatitis were obtained from the Department of Surgery, University Medical Center Halle, Martin-Luther-University Halle-Wittenberg, Germany. The study on human material was approved by the institutional review board of the Medical Faculty of the Martin-Luther-University Halle-Wittenberg and designated in accordance with the Declaration of Helsinki (Approval number: 2019-037). Written informed consent was obtained from all patients.

### 2.2. Isolation and Cultivation of Fibroblasts

CAFs/DAFs from patient specimens were obtained by the outgrowth method, which has been described previously with modifications [12]. Briefly, pancreatic tissue was cut into ca. 20 mm^3^ pieces, seeded in a plate in the presence of Dulbecco’s Modified Eagle’s Medium (DMEM) low glucose and F12, supplemented with 16% fetal bovine serum, 1% penicillin-streptomycin, and 1% amphotericin, and maintained at 37 °C, 5% CO_2_ until fibroblasts grew out. Single-cell RNA sequencing (10X Genomics, Pleasanton, CA, USA) was performed with cells in passage-3.

### 2.3. Single-Cell RNA Sequencing and Bioinformatics Analysis

Isolated CAFs/DAFs were processed with the 10X Genomics Single Cell 3’ v3 Reagent Kit [13]. Samples were processed according to the manufacturer’s user guide (10X Genomics). Final libraries were sequenced with the NextSeq 500 system (Illumina, San Diego, CA, USA) in high-output mode (paired end, 75 base pairs). Single-cell RNA sequencing and bioinformatics analyses were performed with cellranger 6.0.0. Gene expression was quantified using the default 10X Cell Ranger v3 pipeline using the GRCh37/hg19 Reference provided by 10X Genomics. UMI and genes were filtered for each sample and, in general, cells with a minimum number of 2000 genes were kept. Cells containing more than 5% of mitochondrial reads were excluded from downstream analyses. Downstream analyses were performed using the R package Seurat version 4.0. Each scRNA-seq dataset was scaled by library size and log-transformed and for each sample the top 2000 most variable genes were identified. Data were scaled to unit variance and zero mean and the dimensionality of the data was reduced by principal component analysis (PCA) (15 components) and visualized with t-distributed stochastic neighbor embedding (t-SNE) or Uniform Minifold Approximation and Projection (UMAP). Clustering was performed using the Louvain algorithm on the 15 principal components (resolution = 0.5). Cluster-specific markers were identified with the “FindAllMarkers” function.

### 2.4. Immunocytochemistry

Approximately 2.5 × 10^4^ of CAFs/DAFs were seeded on the slides and incubated overnight. PANC-1 cells were used in addition as control for epithelial cell adhesion molecule (EpCAM) staining. Cells were then fixed with 4% PFA and sequentially immunostained with antibodies directed against alpha-smooth muscle actin (α-SMA) (Invitrogen, 701457), KRT19 (Invitrogen, MA5-31977, Waltham, MA, USA), C-X-C motif chemokine 12 (CXCL12) (Invitrogen, PA5-114344), or EpCAM (Dako, M0804).

### 2.5. GEPIA

For gene expression profiling and overall survival analysis, we conducted bioinformatics analysis on the GEPIA platform using the Kaplan–Meier survival plots tool [14], as described previously [15]. Our analysis included 176 to 178 pancreatic cancer samples based on the Cancer Genome Atlas (TCGA) database. Median expression was used as the threshold between high and low expression. *p* < 0.05 was considered statistically significant.

### 2.6. Data Access

Sequencing raw data are available at European Genome-Phenome Archive (EGA) box with the accession number EGAS00001005889.

## 3. Results

### 3.1. Identification of Highly Variable Genes in Disease-Associated Fibroblasts of Pancreatic Cancer and Chronic Pancreatitis Patients

**Figure 1:**
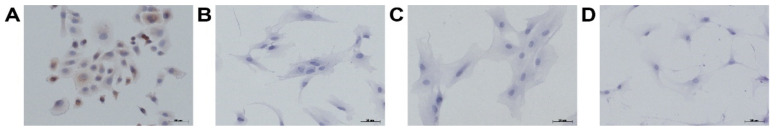
Immunocytochemistry of EpCAM in disease-associated fibroblasts from pancreatic cancer and chronic pancreatitis patients. (**A**) Panc-1 cells stained as positive control, (**B**) cells from patient1, (**C**) from patient2, and (**D**) from patient3. Bar: 50 μm.

**Figure 2:**
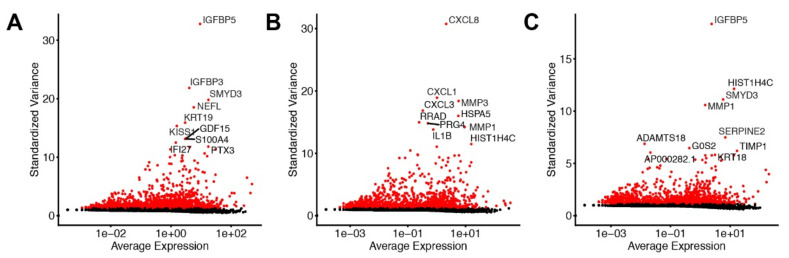
Highly variable features in disease-associated fibroblasts from pancreatic cancer and chronic pancreatitis patients. A subset of features that exhibit high variation between cells in disease-associated fibroblasts (**A**) from patient1, (**B**) from patient2, and (**C**) from patient3.

**Figure 3:**
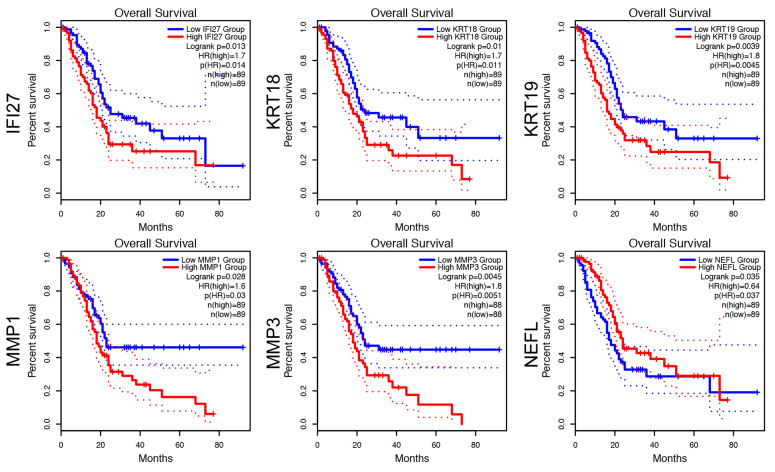
Analysis of 6 genes and overall survival. Overall survival analyses of 6 genes (*IFI27*, *KRT18*, *KRT19*, *MMP1*, *MMP3*, and *NEFL*) in pancreatic cancer based on the GEPIA database. Median expression was used as the threshold between high and low expression. The 95% confidence interval is plotted by the dotted lines.

**Figure 4:**
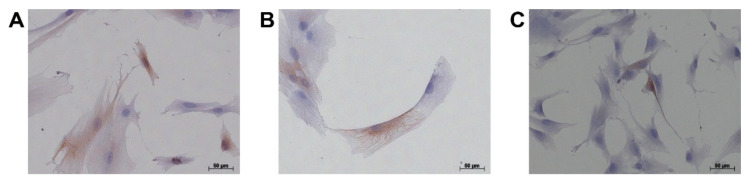
Immunocytochemistry of KRT19 in disease-associated fibroblasts from pancreatic cancer and chronic pancreatitis patients. (**A**) from patient1, (**B**) from patient2, and (**C**) from patient3. Bar: 50 μm.

To study previously described myCAF, iCAF, or apCAF subtypes, and investigate potentially altered CAF states in pancreatic cancer (patient1 and patient2) and chronic pancreatitis patients (patient3) in our cohort (Table 1), we isolated primary disease-associated fibroblasts (DAFs) and performed single-cell RNA sequencing (scRNA-seq) analysis. We selected the outgrowth method, because it is a widely recognized and established method for isolating CAFs. To validate the absence of tumor cells in our samples, we performed immunocytochemistry against EpCAM. We observed positive staining of EpCAM in PANC-1 pancreatic cancer cells, but no positivity was observed in isolated DAFs (Figure 1). After quality control, we obtained 2714, 6764, and 7639 cells, respectively (Table 1). CAFs from patient1 showed several highly variable genes that contribute strongly to cell-to-cell variation, including Insulin Growth Factor Binding Proteins *IGFBP3* and *IGFBP5*, Set- and Mynd Domain Containing 3 (*SMYD3*), Neurofilament Light Chain (*NEFL*), Keratin 19 (*KRT19*), Matrix Metalloprotease 1 (*MMP1*), *MMP3*, metastasis suppressor *KISS1*, Growth Differentiation Factor 15 (*GDF15*), Pentraxin 3 (*PTX3*), S100 Calcium Binding Protein A4 (*S100A4*), and Interferon Alpha Inducible Protein 27 (*IFI27*) (Figure 2A). In CAFs from patient2, we detected chemokines *CXCL1*, *CXCL3*, *CXCL8*, Interleukin 1B (*IL1B*), *MMP1*, *MMP3*, Heat Shock Protein Family A Member 5 (*HSPA5*, also known as GRP78 or BiP), Proteoglycan 4 (*PRG4*), RAS-related Glycolysis Inhibitor And Calcium Channel Regulator (*RRAD*), and *HIST1H4C* (Figure 2B). In the fibroblasts from patient3, we detected *IGFBP5*, *SMYD3*, *MMP1*, *TIMP1*, Serpin Family E Member 2 (*SERPINE2*), *KRT18*, G0/G1 Switch 2 (*G0S2*), ADAM Metallopeptidase with Thrombospondin Type 1 Motif 18 (*ADAMTS18*), and *HIST1H4C* (Figure 2C). To examine whether expression of above mentioned 25 factors is associated with pancreatic cancer patient survival, we performed overall survival (OS) analysis on the GEPIA platform. We identified that expression of *IFI27*, *KRT18*, *KRT19*, *MMP1*, *MMP3*, and *NEFL* are associated with a shorter overall survival of pancreatic cancer patients (Figure 3), whereas the expression of the other 16 genes are not associated with the overall survival of pancreatic cancer patients (not shown). It has been previously shown with a lineage-labeling approach that CAFs of non-PSC origin exhibited elevated expression of *KRT8*, *KRT18*, and *KRT19* [16]. Interestingly, we observed both KRT19-positive and KRT19-negative fibroblasts by immunocytochemistry in all three cases (Figure 4).

### 3.2. Cell Cycle-Associated Factors Are Majority of Highly Variable Genes

**Figure 5:**
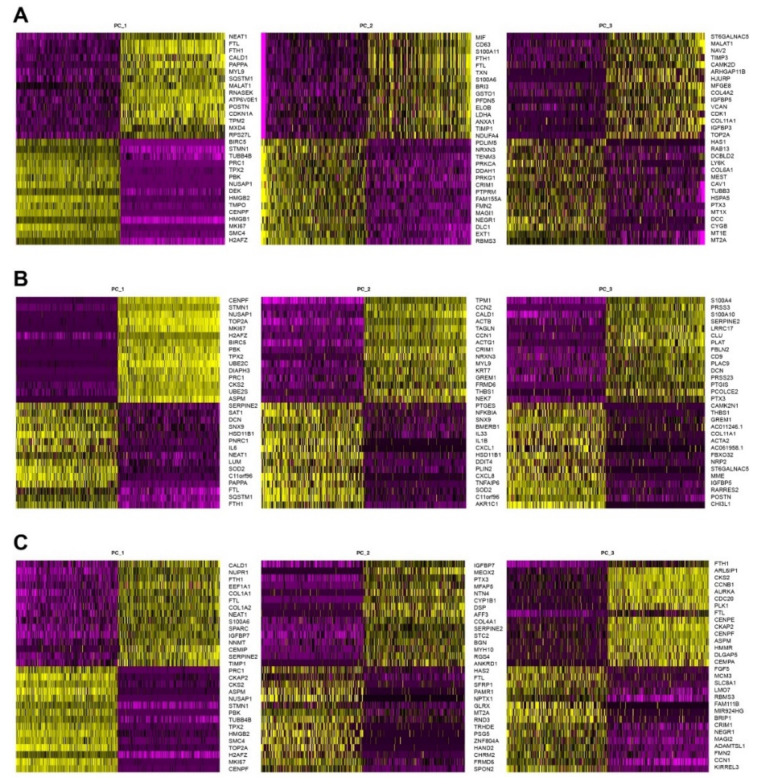
Heat map analyses of scRNAseq data of disease-associated fibroblasts from pancreatic cancer and chronic pancreatitis patients. Top differentially expressed genes were summarized as 3 selected heat map panels in disease-associated fibroblasts (**A**) from patient1, (**B**) from patient2, and (**C**) from patient3.

**Figure 6:**
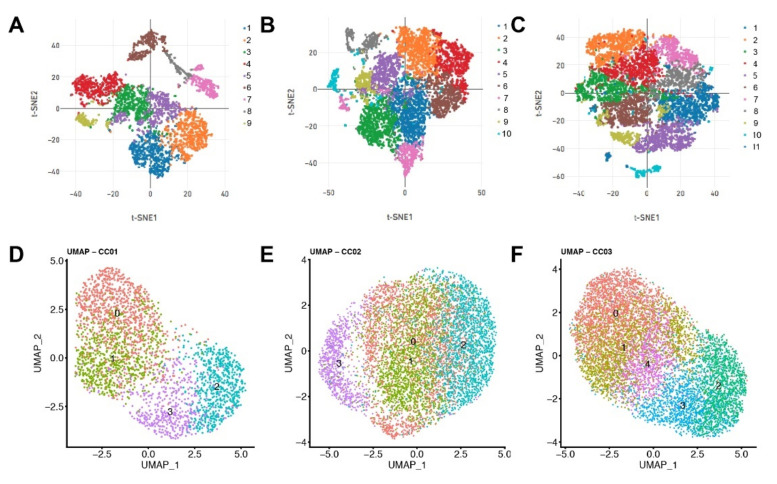
Dimensionality reduction of scRNAseq data. The t-SNE plot analysis of scRNAseq data prior to cell cycle regression (**A**) from patient1, (**B**) from patient2, and (**C**) from patient3. The Uniform Minifold Approximation and Projection (UMAP) plot analysis after cell cycle regression (**D**) from patient1, (**E**) from patient2, and (**F**) from patient3.

Next, to identify which factors are co-expressed together in CAFs/DAFs, we performed heat map analyses and show the top differentially expressed genes in 3 panels for each patient (Figure 5). The analyses revealed that the variance was mostly covered by cell-cycle genes including *MKI67*, *CENPF*, and *CCNB1* (Figure 5). To mitigate the effects of cell cycle heterogeneity in scRNA data, we performed cell cycle regression analysis as previously demonstrated [17]. Prior to cell cycle regression, we observed 9 clusters of cells with similar expression profiles produced by the t-distributed stochastic neighbor embedding (t-SNE) algorithm in CAFs from patient1, 10 clusters in CAFs from patient2, and 11 clusters in fibroblasts from patient3 (Figure 6A–C). After cell cycle regression, we did not observe clearly distinct clusters (Figure 6D–F), suggesting that (1) signals derived from cell cycle-associated factors strongly impact on cluster heterogeneity presentation, and (2) primary isolated and cultured cells are not unique population but exhibit highly similar expression profiling between the clusters. We were still able to divide clusters 0 to 3 in CAFs from patient1, clusters 0 to 3 in CAFs from patient2, as well as clusters 0 to 4 in DAFs from patient3 (Figure 6D–F). We summarize highly variable features after cell cycle regression in Table 2 (patient1), Table 3 (patient2), and Table 4 (patient3). Among 25 highly variable genes identified before cell cycle regression, two factors namely *IGFBP3* and *IGFBP5* were identified as highly variable genes even after cell cycle regression (Table 2, Cluster 0 in Figure 2D), suggesting that these two genes may contribute to CAF heterogeneity independent of cell cycle stage. Interestingly, a number of factors, cell cycle-associated but also non-cell-cycle-associated factors, are identified as highly variable genes shared between CAFs/DAFs isolated from chronic pancreatitis and pancreatic cancer patients (Table 5).

### 3.3. Instead of myCAF-, iCAF-, or apCAF-Specific Clusters, Rather a myCAF-Like Subtype with CXCL12 Expression Observed in Primary Isolated Disease-Associated Fibroblasts

**Figure 7:**
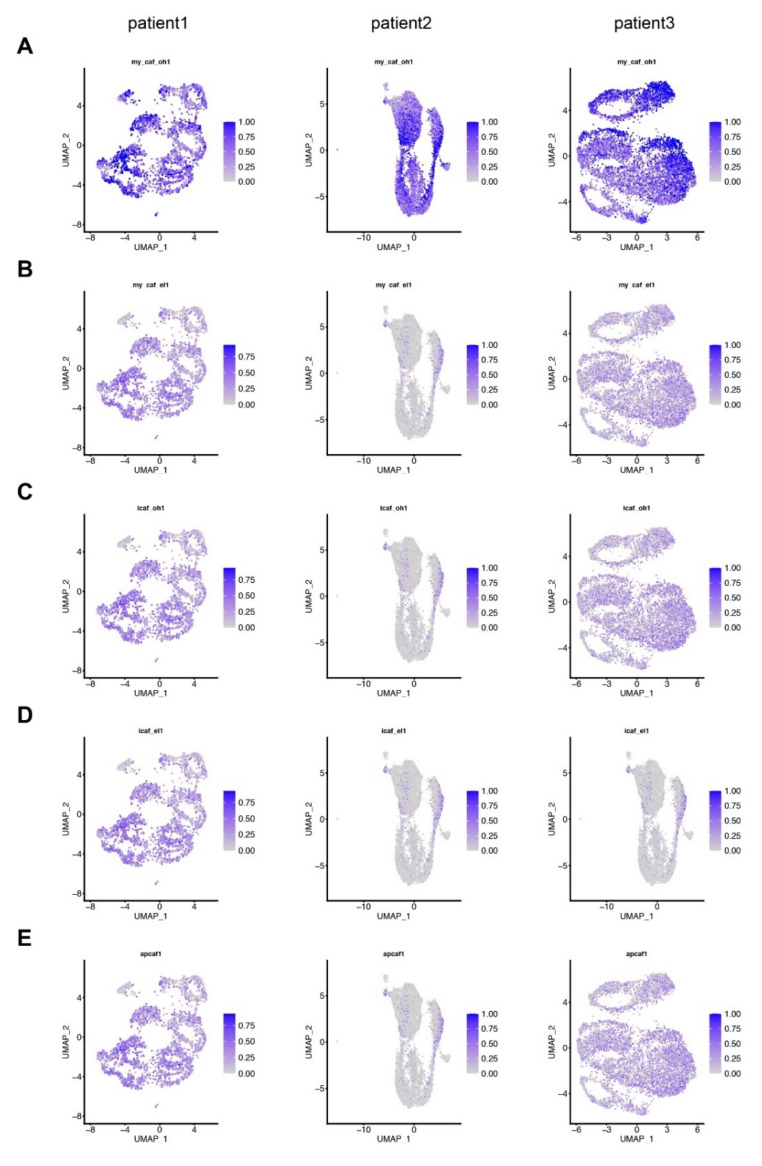
No myofibroblastic, inflammatory or antigen-presenting cancer-associated fibroblast-specific cluster, but exhibit myCAF-like profiling in disease-associated fibroblasts from pancreatic cancer and chronic pancreatitis patients. Cell with (**A**,**B**) myofibroblastic, (**C**,**D**) inflammatory, and (**E**) antigen-presenting cancer-associated fibroblasts are visualized in fibroblasts from pancreatic cancer and chronic pancreatitis patients. Lists of selected markers are listed Table 5, markers were selected from the publications Öhlund et al. [10] for (**A**,**C**), Elyada et al. [11] for (**B**,**D**,**E**).

**Figure 8:**
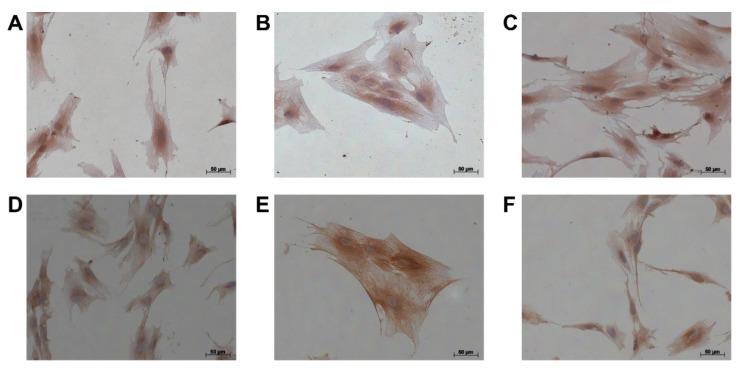
Immunocytochemistry of αSMA and CXCL12 in disease-associated fibroblasts from pancreatic cancer and chronic pancreatitis patients. Cells from (**A**) patient1, (**B**) from patient2, and (**C**) from patient3 for αSMA staining, (**D**) cells from patient1, (**E**) from patient2, and (**F**) from patient3 for CXCL12 staining. Bar: 50 μm.

We next analyzed potential myCAF, iCAF or apCAF transcriptomes in our primary disease-associated fibroblasts from pancreatic cancer and chronic pancreatitis patients. We used myCAF, iCAF, and apCAF markers as previously described [10,11] (see Table 6). As shown in Figure 7, we did not detect any myCAF-, iCAF-, or apCAF-specific cluster in disease-associated fibroblasts from pancreatic cancer or chronic pancreatitis patients. Yet, all disease-associated fibroblasts showed a gene expression profile similar to the myCAFs described by Öhlund et al. (Figure 7A). We observed positive staining of αSMA in all cases, we also observed positive expression of CXCL12, an iCAF marker (Figure 8).

In summary, our single-cell RNA sequencing and bioinformatics analysis identified several highly variable genes including *IFI27*, *KRT18*, *KRT19*, *MMP1*, *MMP3*, and *NEFL*, whose expression is associated with a shorter overall survival of pancreatic cancer patients. The majority of highly variable genes are however dependent on the cell cycle; therefore, we performed cell cycle regression analyses. We identified *IGFBP3* and *IGFBP5* after cell cycle regression, which may potentially contribute to CAF heterogeneity. Here, we could not identify myCAF-, iCAF-, or apCAF-specific clusters, but the expression profiling resemble a rather myCAF-like subtype. Most interestingly, after cell cycle regression, we observed large number of overlapping highly variable genes between samples from pancreatic cancer and chronic pancreatitis patients.

## 4. Discussion

Increasing evidence shows that CAFs represent a highly heterogeneous subpopulations that can be both tumor-promoting and tumor-suppressing, highlighting the importance of the identification and characterization of the diversity of CAF subtypes [4]. In the current study, we isolated primary disease-associated fibroblasts by the outgrowth method from pancreatic cancer and chronic pancreatitis patients. By using single-cell RNA sequencing and bioinformatics analysis, we identified several highly variable genes including *IFI27*, *KRT18*, *KRT19*, *MMP1*, *MMP3,* and *NEFL*. Expression of these genes is associated with shorter overall survival of pancreatic cancer patients (Figure 3). IFI27 has been previously identified as a prognostic marker for pancreatic cancer [18], and involved in pancreatic cancer migration and invasion [19]. The above-mentioned genes are not exclusively expressed in CAFs/DAFs. Yet, our data support that factors expressed in CAFs/DAFs can contribute to pancreatic cancer migration, invasion, and patient outcome.

We selected the outgrowth technique that has been established for isolating pancreatic CAFs and has become a common CAF isolation strategy. In our scRNAseq analysis, we found that the majority of identified highly variable genes are associated with the cell cycle. It was therefore important to conduct cell cycle regression [17], which showed that signals originating from cell cycle-associated factors have a strong impact on cluster heterogeneity (Figure 6). Interestingly, a number of highly variable features after cell cycle regression are commonly found in CAFs/DAFs isolated from chronic pancreatitis and pancreatic cancer patients (Table 5). These 135 factors still include a number of cell cycle-associated factors, but also cell cycle independent factors that are implicated in tumorigenesis. For example, KNSTRN promotes tumorigenesis and gemcitabine resistance in bladder cancer [20]. ZWINT supports pancreatic cancer proliferation [21]. Further studies are required to identify which stromal factors among these 135 genes play a key role in pancreatic cancer. It needs to be further clarified, whether pancreatic cancer-derived CAFs and chronic pancreatitis-derived DAFs have already similar profiling before isolation, or whether they become similar during culture. To that end, it is necessary in the future to establish chronic pancreatitis and pancreatic cancer mouse models with fibroblast lineage-tracing systems, to subsequently isolate CAFs/DAFs, and conduct single-cell RNA sequencing both before and after cell culture. Interestingly, our findings are consistent with the study by Barrera et al. Here, microarray experiments from DAFs isolated by the outgrowth method were performed demonstrating that pancreatic cancer- and chronic pancreatitis-derived fibroblasts share the greatest similarity [7].

One of the goals of the study was to see whether we identify previously described major CAF subtypes, myCAFs-, iCAFs-, and apCAFs. We did not observe any clear myCAFs-, iCAFs-, or apCAFs-specific clusters (Figure 7). Our data are in part consistent with a recent publication, where Grünwald et al. chose the outgrowth method and identified eleven sub-clusters from ten pancreatic cancer patients, but no clear myCAF versus iCAF subpopulation differences [22]. Initially, myCAFs and iCAF subtypes have been described by Öhlund et al. [10]. For the majority of their experiments, quiescent cells were isolated by Collagenase P and DNAse I digestion and co-cultured with pancreatic cancer organoids [10]. Murine late-stage tumors of KPC mice (*Pdx1-Cre; lox-stop-lox-Kras^G12D/+^; Trp53^R172H/+^*) supported the presence of iCAFs and myCAFs, that were isolated by digesting tissue with Pronase, Collagenase P, and DNAse I [23]. Elyada et al. isolated single cells from human pancreatic cancer patients and KPC mice by digestion with Collagenase D, Liberase DL, and DNAse I [11]. Beside myCAFs and iCAFs, the study identified an additional subpopulation of CAFs expressing MHC class II and CD74 (apCAFs) [11]. Notably, in these studies enzymatic digestion methods were applied rather than the outgrowth method for CAF isolation. Whether methodological disparities have an influence on the outcome of these studies, need to be clarified in the future (e.g., via above-mentioned lineage-tracing strategies).

Our study is limited by the small sample size but is the first study to conduct scRNAseq and bioinformatics analyses combined for chronic pancreatitis and pancreatic cancer patients. In summary, our scRNAseq and bioinformatics analysis identified several highly variable genes, some of whose expression is associated with shorter overall survival of pancreatic cancer patients. We could not identify myCAF-, iCAF-, or apCAF-specific clusters, but the expression profiling resemble rather a myCAF-like subtype. Most interestingly, after cell cycle regression, we observed a large number of overlapping highly variable genes between samples from pancreatic cancer and chronic pancreatitis patients, which need to be verified and functionally characterized in both pancreatic cancer and chronic pancreatitis in vivo.

## Figures and Tables

**Table 1:** Information of the patients and analyzed number of single cells.

Title 1	Patient1	Patient2	Patient3
Age at the resection	79	48	25
gender	male	female	male
Disease, Tumor classification	Pancreatic cancer, G2 pT3 pN1 M0, L0 V0 Pn1, UICC-Status IIB	Pancreatic cancer, G2 pT2 pN0 M0, L0 V0 Pn1, UICC-Status IB	Chronic pancreatitis
Number of nuclei	2714	6764	7639

**Table 2:** Highly variable genes after cell cycle regression in CAFs isolated from Patient1.

Gene	Cluster	*p*-Value	Average log2FC	Pct.1	Pct.2	*p*-Value Adjusted
*MXD4*	0	5.03 × 10^−115^	0.52657675335757	0.889	0.508	1.16 × 10^−110^
*PAPPA*	0	9.83 × 10^−86^	1.0109090899262	0.842	0.551	2.27 × 10^−81^
*GDF15*	0	2.41 × 10^−73^	1.16972243694366	0.629	0.281	5.57 × 10^−69^
*CDC42EP5*	0	3.35 × 10^−69^	0.329695153407811	0.651	0.294	7.75 × 10^−65^
*SAT1*	0	7.20 × 10^−64^	0.604752674279049	0.866	0.618	1.66 × 10^−59^
*THBS2*	0	3.09 × 10^−57^	0.420962922589772	0.905	0.656	7.14 × 10^−53^
*CTSK*	0	1.11 × 10^−56^	0.922547366516297	0.713	0.442	2.57 × 10^−52^
*COX7A1*	0	1.63 × 10^−56^	0.481923174187465	0.777	0.502	3.76 × 10^−52^
*SNRPN*	0	3.29 × 10^−55^	0.367555328393367	0.905	0.679	7.62 × 10^−51^
*FADS3*	0	2.22 × 10^−51^	0.372644480541774	0.842	0.593	5.12 × 10^−47^
*PCDH10*	0	3.71 × 10^−50^	0.388362189718403	0.562	0.248	8.58 × 10^−46^
*SCG5*	0	1.47 × 10^−47^	0.897313835683302	0.684	0.414	3.40 × 10^−43^
*IGFBP5*	0	6.23 × 10^−47^	1.89606245760768	0.648	0.388	1.44 × 10^−42^
*AHNAK2*	0	1.58 × 10^−45^	0.342619142825	0.74	0.438	3.66 × 10^−41^
*C5orf46*	0	2.85 × 10^−44^	0.434334936421886	0.602	0.32	6.60 × 10^−40^
*AC097480.1*	0	3.92 × 10^−44^	0.397420295368166	0.376	0.117	9.07 × 10^−41^
*CRYAB*	0	4.14 × 10^−41^	0.719531739217665	0.795	0.554	9.58 × 10^−37^
*IGFBP3*	0	2.10 × 10^−40^	1.30767448451024	0.691	0.435	4.85 × 10^−36^
*ANGPTL4*	0	3.34 × 10^−40^	0.355106223670863	0.543	0.256	7.73 × 10^−36^
*FSTL3*	0	9.44 × 10^−38^	0.332060887611438	0.761	0.491	2.18 × 10^−33^
*COPZ2*	0	2.37 × 10^−37^	0.326611462306643	0.891	0.678	5.48 × 10^−33^
*GPNMB*	0	3.63 × 10^−35^	0.353241339224302	0.761	0.522	8.38 × 10^−31^
*LMCD1*	0	4.55 × 10^−34^	0.505462991451827	0.635	0.395	1.05 × 10^−29^
*LUM*	0	7.07 × 10^−29^	0.407477532269438	0.69	0.454	1.63 × 10^−24^
*COL11A1*	0	8.50 × 10^−27^	0.410716209611568	0.59	0.349	1.96 × 10^−23^
*ANKRD37*	0	1.84 × 10^−26^	0.357421975813258	0.474	0.231	4.26 × 10^−22^
*KIF26B*	0	1.09 × 10^−24^	0.454003954754278	0.697	0.449	2.53 × 10^−19^
*CYP1B1*	0	2.93 × 10^−21^	0.310903502762083	0.473	0.245	6.78 × 10^−17^
*ABI3BP*	0	5.40 × 10^−21^	0.504578102968974	0.402	0.192	1.25 × 10^−16^
*CDK1*	1	3.38 × 10^−281^	1.217773701271	0.923	0.232	7.81 × 10^−277^
*HJURP*	1	8.28 × 10^−214^	0.666094535423477	0.776	0.148	1.91 × 10^−210^
*UBE2C*	1	1.45 × 10^−211^	1.5585090216938	0.937	0.407	3.36 × 10^−207^
*TOP2A*	1	1.19 × 10^−206^	1.54271793537239	0.936	0.435	2.76 × 10^−202^
*AURKB*	1	3.29 × 10^−205^	0.74616142540391	0.847	0.23	7.60 × 10^−201^
*FBXO5*	1	1.02 × 10^−203^	0.67512175613025	0.873	0.249	2.35 × 10^−199^
*HIST1H1B*	1	3.44 × 10^−195^	1.45922164557741	0.777	0.185	7.95 × 10^−191^
*CDCA2*	1	1.35 × 10^−188^	0.692330589773016	0.825	0.222	3.12 × 10^−184^
*KIF18B*	1	6.20 × 10^−188^	0.56145936836675	0.75	0.155	1.43 × 10^−183^
*CDCA8*	1	3.90 × 10^−179^	0.752658739698257	0.837	0.226	9.02 × 10^−175^
*CKAP2L*	1	8.64 × 10^−179^	0.60469745700897	0.821	0.221	2.00 × 10^−174^
*ARHGAP11B*	1	7.96 × 10^−172^	0.550161023821282	0.713	0.152	1.84 × 10^−167^
*CEP128*	1	5.52 × 10^−170^	0.586677178192601	0.82	0.237	1.28 × 10^−165^
*HMGB2*	1	7.70 × 10^−169^	1.24812017681573	0.958	0.53	1.78 × 10^−164^
*LINC01572*	1	9.80 × 10^−155^	0.633946121686711	0.787	0.263	2.27 × 10^−151^
*MELK*	1	3.91 × 10^−151^	0.565960749066164	0.865	0.335	9.05 × 10^−147^
*ESCO2*	1	1.19 × 10^−147^	0.480935013769491	0.754	0.202	2.76 × 10^−143^
*GTSE1*	1	6.96 × 10^−147^	0.727246306246762	0.844	0.317	1.61 × 10^−142^
*NCAPG*	1	8.42 × 10^−147^	0.657784193430886	0.888	0.348	1.95 × 10^−142^
*KIFC1*	1	1.29 × 10^−145^	0.52383057541541	0.803	0.25	2.99 × 10^−140^
*KIF23*	1	2.14 × 10^−143^	0.771038833443001	0.895	0.441	4.95 × 10^−139^
*NEIL3*	1	7.34 × 10^−143^	0.372824444469321	0.626	0.118	1.70 × 10^−139^
*DLEU2*	1	4.85 × 10^−140^	0.628595309288741	0.933	0.5	1.12 × 10^−135^
*KIF11*	1	3.37 × 10^−137^	0.570091739120192	0.833	0.298	7.79 × 10^−133^
*NDC80*	1	5.48 × 10^−136^	0.573873285368215	0.821	0.271	1.27 × 10^−131^
*NSD2*	1	6.78 × 10^−134^	0.683028968844798	0.973	0.604	1.57 × 10^−129^
*UBE2T*	1	2.76 × 10^−133^	0.717575875506757	0.97	0.621	6.38 × 10^−129^
*CDCA5*	1	3.45 × 10^−129^	0.396031325743175	0.706	0.182	7.99 × 10^−125^
*KNL1*	1	2.60 × 10^−127^	0.590067939428105	0.827	0.311	6.02 × 10^−124^
*CENPK*	1	1.68 × 10^−126^	0.595773001526377	0.933	0.489	3.88 × 10^−123^
*BARD1*	1	1.05 × 10^−124^	0.497621617904068	0.847	0.341	2.43 × 10^−120^
*MKI67*	1	3.50 × 10^−124^	0.990647290602104	0.918	0.482	8.09 × 10^−120^
*KIF20B*	1	8.54 × 10^−122^	0.678820111686073	0.915	0.428	1.97 × 10^−117^
*TTK*	1	1.72 × 10^−118^	0.39198516969214	0.633	0.15	3.98 × 10^−114^
*SMC4*	1	3.37 × 10^−118^	0.831072141566686	0.974	0.662	7.79 × 10^−114^
*CDCA4*	1	9.86 × 10^−118^	0.462152550274466	0.818	0.345	2.28 × 10^−113^
*KIF4A*	1	5.43 × 10^−117^	0.506969008189571	0.803	0.299	1.26 × 10^−112^
*FEN1*	1	3.23 × 10^−116^	0.581337824059999	0.91	0.456	7.46 × 10^−112^
*NUF2*	1	4.54 × 10^−116^	0.646107435494053	0.836	0.335	1.05 × 10^−110^
*KIF14*	1	5.91 × 10^−115^	0.463692524219373	0.705	0.212	1.37 × 10^−110^
*DBF4*	1	1.95 × 10^−114^	0.587834146901801	0.858	0.436	4.52 × 10^−110^
*SPC25*	1	1.04 × 10^−113^	0.521305325488545	0.818	0.323	2.40 × 10^−109^
*KIF2C*	1	1.08 × 10^−113^	0.512991064138338	0.727	0.223	2.49 × 10^−109^
*CCNA2*	1	1.33 × 10^−113^	0.58858592921946	0.848	0.337	3.09 × 10^−109^
*FAM83D*	1	1.36 × 10^−114^	0.388259146399628	0.613	0.154	3.14 × 10^−109^
*KIF15*	1	4.23 × 10^−112^	0.308609790169668	0.593	0.134	9.78 × 10^−108^
*HIST1H1E*	1	8.84 × 10^−112^	0.930971071463036	0.719	0.306	2.04 × 10^−107^
*SGO1*	1	1.18 × 10^−111^	0.414278244461817	0.719	0.218	2.73 × 10^−107^
*CLSPN*	1	2.91 × 10^−111^	0.65508914483109	0.956	0.501	6.73 × 10^−107^
*HIST1H2AH*	1	7.15 × 10^−111^	0.375216848297376	0.471	0.077	1.65 × 10^−106^
*TMPO*	1	2.97 × 10^−110^	0.707133223014209	0.973	0.692	6.87 × 10^−106^
*BRIP1*	1	3.94 × 10^−110^	0.610259058781836	0.863	0.392	9.10 × 10^−106^
*HIST1H3B*	1	6.36 × 10^−110^	0.339009976604904	0.492	0.088	1.47 × 10^−105^
*CENPE*	1	2.31 × 10^−109^	0.83868521493331	0.836	0.385	5.35 × 10^−105^
*ATAD2*	1	3.36 × 10^−110^	0.646418548726152	0.944	0.533	7.76 × 10^−105^
*MIR924HG*	1	1.87 × 10^−108^	0.60912425610384	0.734	0.238	4.32 × 10^−104^
*FAM111A*	1	4.28 × 10^−108^	0.480066568863614	0.757	0.321	9.90 × 10^−104^
*POLQ*	1	5.56 × 10^−107^	0.326887174080224	0.619	0.156	1.29 × 10^−102^
*Z94721.1*	1	6.30 × 10^−106^	0.311290952655472	0.568	0.129	1.46 × 10^−101^
*ASPM*	1	6.94 × 10^−106^	0.806476667727835	0.852	0.381	1.60 × 10^−101^
*KIF18A*	1	2.04 × 10^−105^	0.457985114861287	0.764	0.307	4.71 × 10^−101^
*HIST1H3D*	1	6.05 × 10^−105^	0.38895099141706	0.489	0.094	1.40 × 10^−100^
*MIS18BP1*	1	8.74 × 10^−104^	0.543388638803541	0.923	0.59	2.02 × 10^−100^
*NCAPH*	1	9.30 × 10^−104^	0.318441669167983	0.643	0.177	2.15 × 10^−99^
*H2AFX*	1	9.41 × 10^−104^	0.685703332643443	0.94	0.632	2.18 × 10^−99^
*ARHGAP11A*	1	4.07 × 10^−103^	0.432432564600806	0.698	0.243	9.42 × 10^−99^
*TACC3*	1	1.13 × 10^−102^	0.584123376464301	0.865	0.405	2.61 × 10^−98^
*BUB1B*	1	1.58 × 10^−101^	0.450386318125073	0.74	0.257	3.65 × 10^−97^
*CCDC18*	1	1.78 × 10^−101^	0.501883274033725	0.786	0.355	4.11 × 10^−97^
*ANLN*	1	8.20 × 10^−100^	0.686740618692537	0.922	0.505	1.90 × 10^−95^
*RAD51AP1*	1	3.03 × 10^−99^	0.385345488278663	0.758	0.277	7.01 × 10^−95^
*CIT*	1	5.55 × 10^−99^	0.425635672538582	0.678	0.23	1.28 × 10^−94^
*CCNF*	1	3.61 × 10^−97^	0.309720189642715	0.533	0.124	8.35 × 10^−93^
*CENPU*	1	3.42 × 10^−96^	0.517098949441927	0.921	0.482	7.91 × 10^−92^
*USP1*	1	4.73 × 10^−97^	0.529622421208818	0.928	0.59	1.09 × 10^−91^
*ARHGAP11A*	1	4.07 × 10^−103^	0.432432564600806	0.698	0.243	9.42 × 10^−99^
*TACC3*	1	1.13 × 10^−102^	0.584123376464301	0.865	0.405	2.61 × 10^−98^
*BUB1B*	1	1.58 × 10^−101^	0.450386318125073	0.74	0.257	3.65 × 10^−97^
*CCDC18*	1	1.78 × 10^−101^	0.501883274033725	0.786	0.355	4.11 × 10^−97^
*ANLN*	1	8.20 × 10^−100^	0.686740618692537	0.922	0.505	1.90 × 10^−95^
*RAD51AP1*	1	3.03 × 10^−99^	0.385345488278663	0.758	0.277	7.01 × 10^−95^
*CIT*	1	5.55 × 10^−99^	0.425635672538582	0.678	0.23	1.28 × 10^−94^
*CCNF*	1	3.61 × 10^−97^	0.309720189642715	0.533	0.124	8.35 × 10^−93^
*CENPU*	1	3.42 × 10^−96^	0.517098949441927	0.921	0.482	7.91 × 10^−92^
*USP1*	1	4.73 × 10^−97^	0.529622421208818	0.928	0.59	1.09 × 10^−91^
*GAS2L3*	1	5.48 × 10^−96^	0.440448497779858	0.641	0.214	1.27 × 10^−91^
*CENPI*	1	1.43 × 10^−95^	0.313820083241218	0.611	0.176	3.31 × 10^−91^
*RRM2*	1	1.66 × 10^−95^	0.643511970429096	0.945	0.558	3.83 × 10^−91^
*CENPA*	1	8.64 × 10^−95^	0.511135030394721	0.682	0.244	2.00 × 10^−90^
*SKA3*	1	5.87 × 10^−94^	0.324596062484181	0.679	0.221	1.36 × 10^−89^
*ZWINT*	1	1.05 × 10^−92^	0.531799371626574	0.919	0.536	2.43 × 10^−88^
*NUSAP1*	1	1.96 × 10^−92^	0.693560843591611	0.918	0.522	4.53 × 10^−88^
*SGO2*	1	1.41 × 10^−90^	0.580055932148788	0.825	0.394	3.27 × 10^−86^
*SHCBP1*	1	3.79 × 10^−89^	0.448247740254958	0.869	0.404	8.76 × 10^−85^
*DNAJC9*	1	8.51 × 10^−89^	0.519479438857925	0.975	0.77	1.97 × 10^−83^
*ASF1B*	1	1.37 × 10^−87^	0.343159210532476	0.69	0.239	3.16 × 10^−83^
*HIST2H2AC*	1	1.63 × 10^−87^	0.679980454603195	0.723	0.36	3.78 × 10^−83^
*C21orf58*	1	1.34 × 10^−86^	0.308264298086868	0.638	0.203	3.11 × 10^−82^
*BRCA1*	1	4.39 × 10^−86^	0.378210564781065	0.776	0.318	1.02 × 10^−81^
*GMNN*	1	3.35 × 10^−85^	0.483939504250589	0.917	0.56	7.74 × 10^−81^
*ORC6*	1	4.22 × 10^−85^	0.398267082494843	0.801	0.345	9.76 × 10^−81^
*MND1*	1	1.25 × 10^−84^	0.324291743759601	0.679	0.235	2.88 × 10^−81^
*CENPF*	1	1.95 × 10^−83^	0.961460630993616	0.921	0.551	4.50 × 10^−79^
*MXD3*	1	1.44 × 10^−81^	0.36440768070372	0.566	0.177	3.33 × 10^−77^
*RRM1*	1	3.17 × 10^−81^	0.474604869567564	0.94	0.655	7.33 × 10^−77^
*HIST1H1A*	1	1.80 × 10^−80^	0.710605680679022	0.493	0.137	4.16 × 10^−76^
*MAD2L1*	1	5.20 × 10^−80^	0.489488750455522	0.908	0.513	1.20 × 10^−75^
*AURKA*	1	1.24 × 10^−79^	0.63054187607067	0.825	0.42	2.86 × 10^−74^
*ECT2*	1	9.18 × 10^−78^	0.446260241003061	0.837	0.466	2.12 × 10^−73^
*RACGAP1*	1	3.16 × 10^−77^	0.357060353701338	0.732	0.304	7.30 × 10^−73^
*CDC45*	1	2.22 × 10^−76^	0.308840494379532	0.709	0.272	5.14 × 10^−72^
*RFC3*	1	3.01 × 10^−76^	0.343352218848334	0.769	0.321	6.97 × 10^−73^
*PBK*	1	3.17 × 10^−75^	0.567611838947207	0.919	0.499	7.32 × 10^−71^
*PKMYT1*	1	3.47 × 10^−74^	0.306089875792054	0.654	0.235	8.03 × 10^−70^
*BRCA2*	1	1.42 × 10^−73^	0.34965053011471	0.704	0.281	3.28 × 10^−69^
*MYBL1*	1	3.34 × 10^−72^	0.349226656230426	0.682	0.296	7.72 × 10^−68^
*PIF1*	1	8.17 × 10^−72^	0.338413259822857	0.448	0.122	1.89 × 10^−67^
*TPX2*	1	9.93 × 10^−72^	0.664286155940432	0.93	0.605	2.30 × 10^−67^
*FANCI*	1	8.02 × 10^−71^	0.334501015448917	0.798	0.369	1.85 × 10^−66^
*HELLS*	1	2.83 × 10^−70^	0.436294541448265	0.878	0.504	6.55 × 10^−66^
*CENPP*	1	3.61 × 10^−70^	0.429243416895106	0.769	0.386	8.34 × 10^−66^
*PRIM2*	1	9.54 × 10^−70^	0.445663113372904	0.926	0.615	2.21 × 10^−65^
*TCF19*	1	2.05 × 10^−69^	0.306977990083555	0.682	0.265	4.75 × 10^−67^
*LMNB1*	1	1.72 × 10^−68^	0.334939880529808	0.751	0.317	3.99 × 10^−64^
*MIS18A*	1	2.62 × 10^−68^	0.351894600497854	0.77	0.39	6.06 × 10^−64^
*CIP2A*	1	3.52 × 10^−68^	0.35413555558672	0.758	0.351	8.15 × 10^−64^
*NAV2*	1	5.25 × 10^−68^	0.715967019361379	0.907	0.698	1.21 × 10^−63^
*EZH2*	1	9.96 × 10^−68^	0.330376830176061	0.779	0.354	2.30 × 10^−63^
*POLD3*	1	1.03 × 10^−67^	0.346527003387637	0.747	0.358	2.38 × 10^−63^
*SPATA5*	1	1.55 × 10^−67^	0.424438610846798	0.833	0.501	3.58 × 10^−63^
*NCAPG2*	1	6.97 × 10^−67^	0.370094493158939	0.802	0.413	1.61 × 10^−62^
*VRK1*	1	1.58 × 10^−66^	0.350828198823895	0.795	0.411	3.64 × 10^−62^
*SCLT1*	1	2.50 × 10^−66^	0.538567884484186	0.881	0.639	5.78 × 10^−62^
*G2E3*	1	1.41 × 10^−65^	0.434931481714217	0.809	0.518	3.26 × 10^−61^
*HIST1H1C*	1	6.02 × 10^−65^	0.749471025632811	0.792	0.533	1.39 × 10^−60^
*PCLAF*	1	7.63 × 10^−65^	0.548409542540011	0.982	0.726	1.76 × 10^−60^
*CEP55*	1	2.28 × 10^−64^	0.444384877721666	0.883	0.448	5.26 × 10^−60^
*AP001347.1*	1	2.65 × 10^−64^	0.338860490459612	0.697	0.306	6.13 × 10^−60^
*RAD18*	1	3.64 × 10^−62^	0.349511034427767	0.813	0.464	8.42 × 10^−58^
*MYBL2*	1	4.80 × 10^−62^	0.327599270826376	0.701	0.309	1.11 × 10^−57^
*APOLD1*	1	1.04 × 10^−61^	0.482705829625938	0.714	0.356	2.40 × 10^−57^
*PSMC3IP*	1	3.62 × 10^−60^	0.305727970041719	0.775	0.366	8.37 × 10^−56^
*MCM7*	1	5.97 × 10^−59^	0.406425723666096	0.947	0.575	1.38 × 10^−54^
*GPSM2*	1	1.11 × 10^−57^	0.356375237610496	0.653	0.299	2.58 × 10^−53^
*NCAPD3*	1	1.47 × 10^−55^	0.323452379603221	0.81	0.47	3.39 × 10^−51^
*CKAP2*	1	1.48 × 10^−55^	0.549640977439134	0.872	0.602	3.42 × 10^−51^
*CDKN2D*	1	5.12 × 10^−54^	0.441625540592813	0.683	0.387	1.18 × 10^−49^
*SMC2*	1	1.35 × 10^−53^	0.382496233463084	0.93	0.671	3.11 × 10^−49^
*BUB1*	1	1.40 × 10^−53^	0.311722421521002	0.699	0.307	3.24 × 10^−49^
*FOXM1*	1	2.19 × 10^−53^	0.300815092823151	0.75	0.378	5.06 × 10^−50^
*TEX30*	1	3.39 × 10^−51^	0.319135647569659	0.842	0.546	7.84 × 10^−47^
*PRR11*	1	6.78 × 10^−52^	0.383434189793599	0.803	0.444	1.57 × 10^−46^
*GINS2*	1	1.38 × 10^−50^	0.365751194770039	0.885	0.526	3.20 × 10^−45^
*EMP2*	1	5.82 × 10^−51^	0.34924550830157	0.872	0.6	1.34 × 10^−44^
*TRIM59*	1	7.84 × 10^−48^	0.391721961373452	0.772	0.506	1.81 × 10^−43^
*HIST1H1D*	1	3.16 × 10^−47^	0.440065411259682	0.331	0.081	7.30 × 10^−43^
*HMMR*	1	5.48 × 10^−46^	0.406688350508869	0.839	0.441	1.27 × 10^−41^
*SMC1A*	1	2.73 × 10^−45^	0.34372240444355	0.895	0.678	6.31 × 10^−41^
*CCDC14*	1	3.02 × 10^−46^	0.328566007707011	0.801	0.507	6.98 × 10^−41^
*KIF22*	1	5.25 × 10^−45^	0.306036800059592	0.777	0.479	1.21 × 10^−40^
*PLK1*	1	3.34 × 10^−44^	0.417998745939095	0.678	0.336	7.73 × 10^−39^
*LCORL*	1	1.35 × 10^−42^	0.388923389039701	0.776	0.521	3.13 × 10^−38^
*DEPDC1*	1	3.55 × 10^−42^	0.325869872955602	0.742	0.37	8.21 × 10^−38^
*SAC3D1*	1	4.47 × 10^−41^	0.321992720632306	0.895	0.677	1.03 × 10^−36^
*PRC1*	1	5.34 × 10^−40^	0.434659757767406	0.934	0.581	1.23 × 10^−35^
*DHFR*	1	3.09 × 10^−39^	0.315986392775952	0.908	0.686	7.14 × 10^−35^
*CENPM*	1	1.11 × 10^−38^	0.324964822919566	0.906	0.615	2.56 × 10^−34^
*BIRC5*	1	7.70 × 10^−35^	0.40221160065042	0.945	0.655	1.78 × 10^−30^
*RFX8*	1	1.72 × 10^−34^	0.305449206969217	0.654	0.362	3.98 × 10^−29^
*SAP30*	1	6.59 × 10^−31^	0.320896181997167	0.783	0.569	1.52 × 10^−26^
*RAD51B*	1	1.46 × 10^−29^	0.367003320328178	0.813	0.594	3.38 × 10^−24^
*KNSTRN*	1	1.59 × 10^−22^	0.302722797631973	0.764	0.552	3.67 × 10^−18^
*CCNB1*	2	1.38 × 10^−170^	1.28130409189154	0.997	0.65	3.19 × 10^−166^
*CDC20*	2	6.96 × 10^−149^	1.08085310140798	0.975	0.51	1.61 × 10^−144^
*CCNB2*	2	1.08 × 10^−147^	0.79963647553927	0.938	0.428	2.51 × 10^−143^
*DLGAP5*	2	2.02 × 10^−127^	0.776139871899789	0.94	0.427	4.68 × 10^−123^
*PRC1*	2	2.18 × 10^−117^	0.870056869643796	0.987	0.589	5.03 × 10^−113^
*HMMR*	2	3.01 × 10^−114^	0.754271372359569	0.943	0.437	6.95 × 10^−110^
*CDKN3*	2	5.32 × 10^−111^	0.769225621045913	0.993	0.691	1.23 × 10^−106^
*BIRC5*	2	3.17 × 10^−98^	0.735538745956502	0.987	0.662	7.33 × 10^−94^
*CKS1B*	2	2.03 × 10^−94^	0.691059605394196	0.99	0.75	4.70 × 10^−88^
*TPX2*	2	4.90 × 10^−92^	0.80960851990548	0.978	0.612	1.13 × 10^−87^
*CENPF*	2	3.71 × 10^−91^	0.857661764249716	0.992	0.555	8.59 × 10^−87^
*CDCA3*	2	6.47 × 10^−89^	0.545369444372327	0.871	0.419	1.50 × 10^−84^
*PBK*	2	3.82 × 10^−84^	0.6553913393387	0.953	0.517	8.83 × 10^−80^
*DEPDC1*	2	9.19 × 10^−81^	0.562161053568371	0.811	0.374	2.13 × 10^−76^
*CEP55*	2	1.67 × 10^−77^	0.617579552600243	0.891	0.473	3.86 × 10^−74^
*PIMREG*	2	1.84 × 10^−75^	0.469640939018683	0.755	0.352	4.25 × 10^−71^
*PHF19*	2	7.36 × 10^−73^	0.522695499227147	0.946	0.67	1.70 × 10^−68^
*NUSAP1*	2	2.76 × 10^−59^	0.510702502616756	0.943	0.54	6.38 × 10^−55^
*PLK1*	2	1.76 × 10^−58^	0.43795685432261	0.745	0.338	4.06 × 10^−54^
*MAD2L1*	2	3.55 × 10^−54^	0.492908983497593	0.898	0.541	8.21 × 10^−50^
*CKAP2*	2	6.02 × 10^−49^	0.598043842889473	0.864	0.621	1.39 × 10^−44^
*SMC4*	2	1.88 × 10^−47^	0.525458738244071	0.977	0.681	4.34 × 10^−43^
*MKI67*	2	7.10 × 10^−47^	0.460075658603473	0.943	0.503	1.64 × 10^−42^
*ANLN*	2	1.47 × 10^−46^	0.434340895876565	0.921	0.532	3.39 × 10^−42^
*NEK2*	2	1.14 × 10^−45^	0.307580708480862	0.526	0.211	2.63 × 10^−41^
*PRR11*	2	4.65 × 10^−46^	0.368030429515734	0.811	0.465	1.08 × 10^−41^
*BUB1*	2	1.91 × 10^−36^	0.317568378806836	0.657	0.344	4.43 × 10^−32^
*TMPO*	2	5.74 × 10^−34^	0.429872635835613	0.948	0.717	1.33 × 10^−29^
*ASPM*	2	1.36 × 10^−33^	0.396222694173503	0.789	0.429	3.14 × 10^−29^
*TACC3*	2	1.11 × 10^−32^	0.332936107197724	0.801	0.453	2.56 × 10^−28^
*MSH6*	2	2.77 × 10^−31^	0.336669026498678	0.747	0.53	6.40 × 10^−27^
*KNSTRN*	2	9.43 × 10^−30^	0.40640158305418	0.765	0.565	2.18 × 10^−25^
*PCLAF*	2	2.54 × 10^−28^	0.412586221887705	0.987	0.741	5.87 × 10^−24^
*H2AFX*	2	5.65 × 10^−26^	0.345102089485394	0.886	0.667	1.31 × 10^−20^
*CENPE*	2	7.96 × 10^−24^	0.31517477923108	0.757	0.436	1.84 × 10^−19^
*AURKA*	2	1.16 × 10^−21^	0.454990793395169	0.705	0.479	2.68 × 10^−17^
*KIF20B*	2	1.19 × 10^−23^	0.343044424382152	0.781	0.497	2.76 × 10^−17^
*RRM2*	2	1.22 × 10^−22^	0.315412549099177	0.901	0.595	2.81 × 10^−17^
*NUF2*	2	1.01 × 10^−20^	0.31369299247181	0.7	0.405	2.34 × 10^−16^
*MCM7*	2	1.11 × 10^−18^	0.302397358738716	0.844	0.628	2.56 × 10^−14^
*SGO2*	2	5.42 × 10^−16^	0.328146528775139	0.687	0.46	1.25 × 10^−11^

**Table 3:** Highly variable genes after cell cycle regression in CAFs isolated from Patient2.

Gene	Cluster	*p*-Value	Average log2FC	Pct.1	Pct.2	*p*-Value Adjusted
*TOP2A*	0	0	1.79605326374694	0.815	0.233	0
*UBE2C*	0	0	1.63919445211954	0.781	0.211	0
*CENPF*	0	0	1.34876124117104	0.811	0.292	0
*CDK1*	0	0	1.33264666202355	0.774	0.138	0
*STMN1*	0	0	1.32138682451592	0.942	0.547	0
*NUSAP1*	0	0	1.2286980735027	0.792	0.238	0
*DIAPH3*	0	0	1.16113907654703	0.851	0.295	0
*CKS2*	0	0	1.15293101811774	0.922	0.62	0
*HMGB2*	0	0	1.11538535017322	0.768	0.246	0
*TYMS*	0	0	1.10000618998348	0.866	0.314	0
*MKI67*	0	0	1.07580673615101	0.731	0.204	0
*SMC4*	0	0	0.985921598378538	0.846	0.44	0
*PCLAF*	0	0	0.968323819792444	0.882	0.296	0
*TPX2*	0	0	0.924358894514124	0.769	0.266	0
*ASPM*	0	0	0.897670249420366	0.678	0.182	0
*CLSPN*	0	0	0.887171912927092	0.757	0.176	0
*ATAD2*	0	0	0.874751526379846	0.775	0.237	0
*PBK*	0	0	0.855784944466262	0.735	0.22	0
*CENPU*	0	0	0.835117757098471	0.782	0.196	0
*CKS1B*	0	0	0.833435203727637	0.876	0.454	0
*UBE2T*	0	0	0.825964540809045	0.785	0.275	0
*RRM2*	0	0	0.82406183500547	0.663	0.161	0
*KIF20B*	0	0	0.815776478647066	0.736	0.207	0
*APOLD1*	0	0	0.797140087161004	0.603	0.156	0
*CENPK*	0	0	0.793137975019939	0.805	0.261	0
*CENPE*	0	0	0.792614984666222	0.673	0.19	0
*TACC3*	0	0	0.766915247357903	0.705	0.199	0
*TMPO*	0	0	0.739947410344141	0.75	0.267	0
*ANLN*	0	0	0.732349867027408	0.691	0.206	0
*SGO2*	0	0	0.729181104940837	0.693	0.209	0
*TK1*	0	0	0.724386338545294	0.783	0.238	0
*HMMR*	0	0	0.699172842546925	0.645	0.161	0
*NCAPG*	0	0	0.697319371070576	0.645	0.124	0
*FEN1*	0	0	0.696892668232285	0.706	0.197	0
*SPC25*	0	0	0.681763327564178	0.635	0.128	0
*HJURP*	0	0	0.679793595290009	0.556	0.086	0
*FBXO5*	0	0	0.671620101318795	0.611	0.113	0
*NUF2*	0	0	0.640912005859067	0.611	0.148	0
*HELLS*	0	0	0.63451507811957	0.647	0.17	0
*NDC80*	0	0	0.621413114006706	0.616	0.141	0
*GTSE1*	0	0	0.619982966600172	0.6	0.145	0
*AURKB*	0	0	0.606884764450141	0.558	0.092	0
*KIF23*	0	0	0.602477769303273	0.605	0.145	0
*ZWINT*	0	0	0.598416502953059	0.656	0.175	0
*KNL1*	0	0	0.583367784243798	0.584	0.127	0
*KIF11*	0	0	0.572709771587086	0.578	0.131	0
*CDCA8*	0	0	0.538516599881907	0.512	0.089	0
*CKAP2L*	0	0	0.508486954585748	0.524	0.096	0
*CEP128*	0	0	0.485831827254704	0.5	0.086	0
*MELK*	0	0	0.482259210927512	0.538	0.106	0
*ESCO2*	0	4.69 × 10^−295^	0.443942217336555	0.469	0.073	1.12 × 10^−289^
*PSMC3IP*	0	2.56 × 10^−293^	0.534713822780204	0.612	0.161	6.12 × 10^−289^
*AURKA*	0	5.53 × 10^−288^	0.69848370289414	0.62	0.164	1.32 × 10^−283^
*NSD2*	0	6.07 × 10^−287^	0.672994436541658	0.719	0.268	1.45 × 10^−282^
*BRIP1*	0	1.73 × 10^−286^	0.500689898963752	0.469	0.078	4.15 × 10^−282^
*MXD3*	0	3.37 × 10^−286^	0.52543794412312	0.488	0.092	8.08 × 10^−282^
*CEP55*	0	1.45 × 10^−282^	0.626250526855352	0.662	0.187	3.46 × 10^−278^
*ARHGAP11B*	0	5.82 × 10^−282^	0.432470373138973	0.426	0.06	1.39 × 10^−277^
*KIF14*	0	6.53 × 10^−279^	0.458459893320424	0.479	0.085	1.56 × 10^−275^
*KPNA2*	0	6.13 × 10^−277^	1.11884776918933	0.869	0.555	1.47 × 10^−272^
*RFC3*	0	8.41 × 10^−277^	0.468635621907317	0.57	0.137	2.01 × 10^−272^
*CCNA2*	0	1.27 × 10^−276^	0.601184117702451	0.581	0.146	3.04 × 10^−273^
*H2AFX*	0	4.76 × 10^−275^	0.772913382345842	0.734	0.314	1.14 × 10^−270^
*BIRC5*	0	1.80 × 10^−274^	0.811277206411767	0.767	0.27	4.31 × 10^−269^
*CENPM*	0	3.93 × 10^−273^	0.542434709391609	0.67	0.202	9.40 × 10^−269^
*CENPW*	0	1.75 × 10^−272^	0.72148469926098	0.816	0.345	4.20 × 10^−268^
*HIST1H1B*	0	1.11 × 10^−271^	0.707208911930734	0.41	0.059	2.66 × 10^−268^
*PRC1*	0	2.52 × 10^−271^	0.745506641367581	0.746	0.261	6.03 × 10^−268^
*PCNA*	0	2.34 × 10^−270^	0.718422723531482	0.809	0.395	5.60 × 10^−266^
*SHCBP1*	0	1.48 × 10^−268^	0.451644802204119	0.559	0.129	3.55 × 10^−264^
*DNAJC9*	0	9.37 × 10^−267^	0.633949672086213	0.781	0.347	2.25 × 10^−262^
*KIFC1*	0	3.26 × 10^−266^	0.41048060928204	0.478	0.089	7.80 × 10^−262^
*DTL*	0	9.05 × 10^−265^	0.404951277172161	0.428	0.068	2.17 × 10^−260^
*RAD51AP1*	0	2.77 × 10^−264^	0.417840591287033	0.478	0.092	6.64 × 10^−260^
*GINS2*	0	6.91 × 10^−264^	0.460387044513258	0.512	0.11	1.65 × 10^−259^
*CDCA5*	0	7.00 × 10^−262^	0.347587617602858	0.409	0.058	1.68 × 10^−257^
*KIF18B*	0	3.05 × 10^−261^	0.382458042816294	0.404	0.057	7.30 × 10^−257^
*BARD1*	0	8.79 × 10^−260^	0.424084384025479	0.462	0.086	2.11 × 10^−255^
*SGO1*	0	8.35 × 10^−259^	0.41290078141843	0.472	0.09	2.00 × 10^−254^
*KIF4A*	0	3.33 × 10^−255^	0.472499859356929	0.5	0.109	7.97 × 10^−251^
*TTK*	0	7.37 × 10^−255^	0.366775451612081	0.419	0.066	1.76 × 10^−250^
*DTYMK*	0	1.18 × 10^−254^	0.7026225521664	0.913	0.593	2.82 × 10^−250^
*ORC6*	0	9.10 × 10^−254^	0.43159577464565	0.53	0.126	2.18 × 10^−249^
*LINC01572*	0	4.59 × 10^−252^	0.424717002777551	0.429	0.074	1.10 × 10^−247^
*MAD2L1*	0	6.58 × 10^−252^	0.577304939125178	0.699	0.247	1.57 × 10^−247^
*MYBL1*	0	2.37 × 10^−249^	0.365784421616595	0.415	0.066	5.67 × 10^−245^
*CKAP2*	0	1.65 × 10^−248^	0.745562712544766	0.747	0.31	3.95 × 10^−244^
*KIF2C*	0	6.83 × 10^−246^	0.436136580958906	0.456	0.09	1.64 × 10^−241^
*NEIL3*	0	1.20 × 10^−245^	0.346115986129678	0.377	0.051	2.88 × 10^−241^
*SMC2*	0	8.54 × 10^−243^	0.608875725169878	0.821	0.411	2.05 × 10^−238^
*MCM7*	0	1.14 × 10^−241^	0.493220488397421	0.652	0.213	2.73 × 10^−237^
*BRCA1*	0	7.42 × 10^−236^	0.422694688818054	0.509	0.124	1.78 × 10^−231^
*DLGAP5*	0	2.32 × 10^−235^	0.584637912644874	0.618	0.185	5.56 × 10^−231^
*DEPDC1*	0	2.39 × 10^−235^	0.497867650448442	0.574	0.156	5.73 × 10^−231^
*RRM1*	0	3.05 × 10^−235^	0.531960719218836	0.724	0.292	7.30 × 10^−231^
*DNMT1*	0	7.81 × 10^−235^	0.610218771091325	0.854	0.474	1.87 × 10^−230^
*FANCI*	0	5.13 × 10^−233^	0.41420243743029	0.521	0.133	1.23 × 10^−228^
*CENPH*	0	1.86 × 10^−232^	0.448572767362875	0.642	0.207	4.46 × 10^−228^
*BUB1B*	0	3.53 × 10^−231^	0.415512381900352	0.481	0.108	8.46 × 10^−227^
*CDC25C*	0	5.81 × 10^−231^	0.380566646887002	0.403	0.07	1.39 × 10^−226^
*NCAPH*	0	1.11 × 10^−229^	0.318274751878298	0.391	0.063	2.66 × 10^−226^
*CENPX*	0	1.39 × 10^−226^	0.639373459416858	0.922	0.651	3.32 × 10^−222^
*PHF19*	0	5.15 × 10^−226^	0.541201400054887	0.696	0.262	1.23 × 10^−221^
*NCAPG2*	0	3.70 × 10^−224^	0.431846455633852	0.539	0.149	8.86 × 10^−220^
*KIF18A*	0	1.53 × 10^−223^	0.421064420210407	0.48	0.114	3.67 × 10^−219^
*CDCA2*	0	2.31 × 10^−223^	0.449341137619628	0.506	0.133	5.54 × 10^−219^
*PRR11*	0	1.29 × 10^−221^	0.487110880247023	0.561	0.161	3.08 × 10^−217^
*E2F1*	0	2.84 × 10^−221^	0.39788386826894	0.473	0.11	6.81 × 10^−217^
*CENPN*	0	6.53 × 10^−220^	0.480054472791842	0.67	0.246	1.56 × 10^−215^
*CDCA3*	0	3.89 × 10^−218^	0.562071082533152	0.59	0.185	9.32 × 10^−214^
*DLEU2*	0	7.17 × 10^−216^	0.496516580212333	0.623	0.218	1.72 × 10^−210^
*GMNN*	0	2.80 × 10^−214^	0.558870724562077	0.729	0.319	6.71 × 10^−210^
*CCDC18*	0	3.73 × 10^−214^	0.468051889873288	0.558	0.173	8.93 × 10^−210^
*CDC45*	0	1.24 × 10^−213^	0.315790294359164	0.383	0.066	2.97 × 10^−209^
*PTTG1*	0	6.60 × 10^−213^	0.777391296315842	0.881	0.517	1.58 × 10^−208^
*RPL39L*	0	6.71 × 10^−213^	0.557396767350297	0.729	0.327	1.61 × 10^−208^
*MND1*	0	1.89 × 10^−211^	0.315714589477428	0.37	0.061	4.52 × 10^−207^
*ITGB3BP*	0	3.64 × 10^−210^	0.516068351304291	0.726	0.318	8.73 × 10^−206^
*BRCA2*	0	9.28 × 10^−211^	0.342272583284152	0.442	0.097	2.22 × 10^−206^
*SPDL1*	0	3.71 × 10^−209^	0.615851855050538	0.826	0.47	8.89 × 10^−205^
*RACGAP1*	0	2.33 × 10^−204^	0.402972574424527	0.5	0.136	5.58 × 10^−200^
*SKA2*	0	3.70 × 10^−203^	0.538645990835765	0.836	0.455	8.87 × 10^−199^
*CENPA*	0	1.63 × 10^−202^	0.492527034567137	0.513	0.149	3.91 × 10^−198^
*BUB1*	0	4.01 × 10^−202^	0.357204472978074	0.459	0.109	9.60 × 10^−198^
*ARHGAP11A*	0	1.47 × 10^−199^	0.353884246564175	0.422	0.093	3.53 × 10^−196^
*NASP*	0	7.93 × 10^−199^	0.579507057462542	0.882	0.54	1.90 × 10^−194^
*TMEM106C*	0	2.65 × 10^−199^	0.555492375381984	0.771	0.397	6.35 × 10^−194^
*CDKN3*	0	3.21 × 10^−198^	0.64734439585374	0.715	0.284	7.69 × 10^−194^
*VRK1*	0	4.26 × 10^−197^	0.380498837312631	0.538	0.167	1.02 × 10^−192^
*USP1*	0	3.68 × 10^−196^	0.487231052109189	0.682	0.284	8.82 × 10^−192^
*PKMYT1*	0	2.42 × 10^−195^	0.322695046925214	0.386	0.076	5.79 × 10^−191^
*CIT*	0	2.76 × 10^−194^	0.36227876018763	0.412	0.091	6.61 × 10^−190^
*TCF19*	0	3.14 × 10^−194^	0.326812235523065	0.392	0.081	7.51 × 10^−190^
*PLK1*	0	5.72 × 10^−193^	0.478566405086608	0.487	0.136	1.37 × 10^−188^
*DBF4*	0	7.76 × 10^−193^	0.476243325691938	0.579	0.205	1.86 × 10^−189^
*MCM5*	0	4.10 × 10^−188^	0.389797623180505	0.508	0.151	9.83 × 10^−184^
*EMP2*	0	5.79 × 10^−188^	0.482573581188507	0.729	0.328	1.39 × 10^−183^
*CCNB2*	0	9.87 × 10^−187^	0.483990292844758	0.604	0.205	2.36 × 10^−182^
*FOXM1*	0	1.36 × 10^−185^	0.347433950574551	0.483	0.132	3.25 × 10^−181^
*CDKN2C*	0	5.29 × 10^−185^	0.390740413257006	0.524	0.165	1.27 × 10^−180^
*CENPP*	0	1.73 × 10^−184^	0.418338769529916	0.521	0.164	4.14 × 10^−180^
*KNSTRN*	0	2.07 × 10^−183^	0.520280464541216	0.606	0.234	4.96 × 10^−179^
*PRIM2*	0	4.38 × 10^−183^	0.499396287232664	0.702	0.329	1.05 × 10^−178^
*CIP2A*	0	1.15 × 10^−182^	0.338827439703043	0.444	0.115	2.76 × 10^−179^
*C21orf58*	0	3.86 × 10^−183^	0.309679968228961	0.397	0.089	9.25 × 10^−178^
*GAS2L3*	0	3.93 × 10^−182^	0.355561254043055	0.383	0.083	9.42 × 10^−178^
*CDC20*	0	4.93 × 10^−181^	0.524729916423815	0.626	0.221	1.18 × 10^−175^
*RPA3*	0	3.54 × 10^−179^	0.508784005186509	0.849	0.518	8.47 × 10^−175^
*PSIP1*	0	4.21 × 10^−178^	0.531844560159837	0.858	0.525	1.01 × 10^−173^
*CBX5*	0	3.40 × 10^−176^	0.526371278230042	0.849	0.522	8.15 × 10^−173^
*MIS18BP1*	0	4.87 × 10^−175^	0.483623163399267	0.682	0.316	1.17 × 10^−171^
*CCNB1*	0	8.93 × 10^−174^	0.628253981701828	0.691	0.295	2.14 × 10^−169^
*DHFR*	0	9.04 × 10^−174^	0.426468651928734	0.635	0.258	2.17 × 10^−169^
*EZH2*	0	7.76 × 10^−173^	0.317640681430593	0.407	0.099	1.86 × 10^−168^
*NCAPD3*	0	4.50 × 10^−171^	0.360000013437414	0.518	0.171	1.08 × 10^−167^
*CDC6*	0	5.25 × 10^−171^	0.30306426739638	0.343	0.067	1.26 × 10^−166^
*FAM111A*	0	1.10 × 10^−169^	0.391990222575551	0.51	0.172	2.63 × 10^−165^
*CDCA4*	0	2.26 × 10^−167^	0.370946302210805	0.484	0.154	5.41 × 10^−163^
*MIR924HG*	0	5.76 × 10^−166^	0.321676526418005	0.286	0.043	1.38 × 10^−161^
*BUB3*	0	1.56 × 10^−165^	0.575824912784537	0.849	0.575	3.74 × 10^−161^
*CENPQ*	0	5.28 × 10^−165^	0.33197679927466	0.456	0.133	1.27 × 10^−161^
*CDKN2D*	0	1.41 × 10^−156^	0.570905800968053	0.664	0.335	3.37 × 10^−152^
*CHAF1A*	0	7.21 × 10^−156^	0.31441445649264	0.412	0.113	1.73 × 10^−151^
*SAC3D1*	0	1.94 × 10^−155^	0.473418133474899	0.702	0.362	4.65 × 10^−151^
*NAV2*	0	4.38 × 10^−155^	0.685637322475529	0.884	0.663	1.05 × 10^−150^
*POLD3*	0	2.41 × 10^−153^	0.403682002890545	0.559	0.218	5.76 × 10^−149^
*HIST1H1A*	0	5.94 × 10^−153^	0.469000700324917	0.311	0.062	1.42 × 10^−148^
*CCDC34*	0	5.20 × 10^−151^	0.440947201576789	0.689	0.342	1.24 × 10^−146^
*LRR1*	0	2.48 × 10^−150^	0.372292987167317	0.621	0.267	5.95 × 10^−146^
*ECT2*	0	2.06 × 10^−146^	0.369035453280601	0.56	0.221	4.92 × 10^−142^
*RAD21*	0	8.57 × 10^−144^	0.495144592786876	0.804	0.508	2.05 × 10^−139^
*PARP1*	0	4.44 × 10^−139^	0.406065423637836	0.649	0.306	1.06 × 10^−134^
*TUBG1*	0	6.03 × 10^−139^	0.433334255101158	0.778	0.459	1.45 × 10^−134^
*FOSL1*	0	4.94 × 10^−137^	0.491250510826917	0.778	0.485	1.18 × 10^−133^
*SRSF2*	0	5.02 × 10^−138^	0.46724198469059	0.912	0.681	1.20 × 10^−132^
*EXOSC8*	0	1.27 × 10^−136^	0.415536195334573	0.727	0.401	3.05 × 10^−132^
*RFC4*	0	2.50 × 10^−136^	0.358148003209805	0.541	0.218	5.98 × 10^−134^
*CMC2*	0	1.98 × 10^−129^	0.453658147952604	0.853	0.606	4.74 × 10^−125^
*TRIM59*	0	6.80 × 10^−128^	0.369853501293669	0.497	0.195	1.63 × 10^−123^
*NCEH1*	0	9.76 × 10^−126^	0.365913285724998	0.518	0.212	2.34 × 10^−121^
*MCM3*	0	2.65 × 10^−125^	0.375861362978072	0.518	0.214	6.34 × 10^−121^
*SMC1A*	0	8.46 × 10^−125^	0.427372190793345	0.75	0.44	2.03 × 10^−120^
*TEX30*	0	2.02 × 10^−124^	0.370736140970149	0.619	0.295	4.84 × 10^−120^
*NUDT1*	0	9.64 × 10^−123^	0.393853592948846	0.741	0.419	2.31 × 10^−118^
*CSE1L*	0	1.75 × 10^−121^	0.334689815978467	0.615	0.288	4.19 × 10^−117^
*SCLT1*	0	5.47 × 10^−121^	0.459845797339858	0.668	0.359	1.31 × 10^−116^
*ALYREF*	0	8.72 × 10^−121^	0.402862238077345	0.779	0.493	2.09 × 10^−115^
*HAT1*	0	9.02 × 10^−118^	0.38773725726055	0.734	0.422	2.16 × 10^−113^
*SMC3*	0	6.38 × 10^−116^	0.436689045824374	0.836	0.579	1.53 × 10^−111^
*KIRREL3*	0	1.41 × 10^−114^	0.452577593279448	0.662	0.357	3.37 × 10^−110^
*BCL2L12*	0	3.60 × 10^−114^	0.332916074952397	0.561	0.254	8.62 × 10^−110^
*GPSM2*	0	4.01 × 10^−114^	0.322807074183221	0.482	0.194	9.59 × 10^−109^
*RAB3B*	0	2.04 × 10^−112^	0.419372269808872	0.807	0.496	4.88 × 10^−108^
*CDC25B*	0	4.30 × 10^−111^	0.335344921243839	0.477	0.193	1.03 × 10^−106^
*WDR34*	0	1.19 × 10^−110^	0.320815379473183	0.587	0.272	2.85 × 10^−106^
*RFX8*	0	6.64 × 10^−107^	0.328403138105245	0.4	0.142	1.59 × 10^−102^
*G2E3*	0	5.74 × 10^−106^	0.373888532232132	0.578	0.284	1.37 × 10^−101^
*CKAP5*	0	9.76 × 10^−106^	0.389144262518308	0.692	0.397	2.34 × 10^−101^
*EXOSC9*	0	4.42 × 10^−105^	0.335587033772145	0.634	0.325	1.06 × 10^−99^
*EZR*	0	2.08 × 10^−100^	0.3749120699691	0.685	0.385	4.99 × 10^−97^
*ANP32E*	0	1.16 × 10^−99^	0.423805784666755	0.77	0.514	2.79 × 10^−95^
*KIF22*	0	1.84 × 10^−99^	0.389035285574824	0.718	0.43	4.41 × 10^−95^
*FUS*	0	1.36 × 10^−98^	0.390642972327249	0.891	0.691	3.26 × 10^−93^
*RBMX*	0	3.48 × 10^−97^	0.380013251798135	0.846	0.611	8.33 × 10^−93^
*HSPB11*	0	5.70 × 10^−97^	0.375582585560417	0.878	0.648	1.37 × 10^−93^
*PLAU*	0	2.59 × 10^−95^	0.484438089791465	0.536	0.261	6.20 × 10^−90^
*CCDC14*	0	2.89 × 10^−94^	0.313033819300279	0.549	0.267	6.92 × 10^−90^
*ACYP1*	0	4.27 × 10^−94^	0.317264238978913	0.618	0.327	1.02 × 10^−89^
*BTG3*	0	5.53 × 10^−94^	0.412283557307453	0.824	0.576	1.33 × 10^−89^
*HMGB3*	0	2.40 × 10^−92^	0.332437957934013	0.588	0.297	5.76 × 10^−88^
*ARHGAP29*	0	1.91 × 10^−91^	0.331087035035293	0.465	0.203	4.57 × 10^−87^
*PGP*	0	5.01 × 10^−91^	0.363745280592072	0.863	0.642	1.20 × 10^−86^
*POP7*	0	5.29 × 10^−84^	0.35041100976186	0.824	0.595	1.27 × 10^−79^
*MOK*	0	3.21 × 10^−83^	0.46737513519966	0.634	0.38	7.69 × 10^−79^
*CBX1*	0	6.14 × 10^−83^	0.352701885583383	0.844	0.629	1.47 × 10^−75^
*FABP5*	0	1.85 × 10^−78^	0.306117925413734	0.582	0.308	4.43 × 10^−74^
*SAP30*	0	8.53 × 10^−77^	0.352635521735067	0.743	0.504	2.04 × 10^−72^
*MAP3K20*	0	2.57 × 10^−74^	0.33640702090946	0.789	0.545	6.15 × 10^−70^
*HIST1H1E*	0	3.63 × 10^−73^	0.339306408869152	0.367	0.157	8.69 × 10^−69^
*LSM2*	0	2.41 × 10^−70^	0.31840562333533	0.83	0.606	5.77 × 10^−66^
*PBX3*	0	3.22 × 10^−69^	0.370031925013218	0.721	0.488	7.72 × 10^−65^
*HYI*	0	4.02 × 10^−68^	0.320373254026447	0.787	0.55	9.63 × 10^−64^
*WNT5B*	0	3.78 × 10^−66^	0.318356399985377	0.6	0.348	9.06 × 10^−62^
*CRIM1*	0	9.71 × 10^−65^	0.366441919630305	0.82	0.601	2.33 × 10^−61^
*FAF1*	0	4.79 × 10^−63^	0.326595080517033	0.757	0.517	1.15 × 10^−59^
*RBBP7*	0	4.80 × 10^−63^	0.312026848537851	0.761	0.535	1.15 × 10^−58^
*CCDC88A*	0	2.20 × 10^−62^	0.326290677753468	0.759	0.536	5.27 × 10^−58^
*HAS2*	0	6.61 × 10^−54^	0.348787908529369	0.765	0.513	1.58 × 10^−49^
*C11orf96*	2	6.44 × 10^−104^	0.740866964874605	0.754	0.515	1.54 × 10^−99^
*PTTG1*	3	0	1.68818151464034	0.984	0.579	0
*CDKN3*	3	0	1.32280170053098	0.928	0.345	0
*BIRC5*	3	0	1.24448294135578	0.969	0.347	0
*CCNB1*	3	1.68 × 10^−270^	1.53989120433647	0.889	0.351	4.02 × 10^−266^
*CDC20*	3	5.47 × 10^−235^	1.19385593452449	0.806	0.281	1.31 × 10^−230^
*PRC1*	3	1.56 × 10^−225^	1.03131500977058	0.89	0.343	3.73 × 10^−221^
*CCNB2*	3	8.32 × 10^−206^	0.89505539193741	0.772	0.266	1.99 × 10^−201^
*CENPW*	3	1.90 × 10^−196^	0.906748625989908	0.918	0.429	4.55 × 10^−192^
*CENPF*	3	1.65 × 10^−191^	1.10946534906002	0.949	0.382	3.95 × 10^−187^
*DLGAP5*	3	2.36 × 10^−182^	0.828327618951242	0.755	0.258	5.66 × 10^−178^
*STMN1*	3	1.16 × 10^−172^	1.04854350049214	0.993	0.623	2.79 × 10^−168^
*KIF20A*	3	6.24 × 10^−153^	0.478111913734825	0.457	0.109	1.49 × 10^−148^
*PBK*	3	6.93 × 10^−153^	0.768087459409072	0.817	0.317	1.66 × 10^−148^
*TROAP*	3	3.95 × 10^−143^	0.535024517798047	0.575	0.183	9.45 × 10^−139^
*CKS1B*	3	1.16 × 10^−141^	0.749209886022839	0.932	0.535	2.79 × 10^−137^
*CDCA3*	3	9.83 × 10^−139^	0.655516371526958	0.696	0.256	2.35 × 10^−134^
*HMGB3*	3	2.65 × 10^−134^	0.686178765495008	0.733	0.338	6.34 × 10^−130^
*CEP55*	3	2.18 × 10^−129^	0.712781591135284	0.702	0.281	5.23 × 10^−125^
*PHF19*	3	8.56 × 10^−124^	0.626704147754039	0.755	0.345	2.05 × 10^−119^
*ANLN*	3	1.05 × 10^−123^	0.620871330821275	0.751	0.3	2.52 × 10^−119^
*SNHG3*	3	2.41 × 10^−122^	0.614603665812925	0.62	0.264	5.77 × 10^−118^
*ARL6IP1*	3	3.38 × 10^−119^	0.952333640124179	0.899	0.651	8.10 × 10^−116^
*DTYMK*	3	2.31 × 10^−117^	0.677527425601535	0.94	0.657	5.54 × 10^−113^
*LSM5*	3	5.07 × 10^−114^	0.624023436144164	0.941	0.724	1.21 × 10^−109^
*PCLAF*	3	1.06 × 10^−110^	0.634544335405642	0.878	0.42	2.53 × 10^−107^
*DEPDC1*	3	1.15 × 10^−109^	0.638099370253651	0.611	0.239	2.76 × 10^−104^
*MAD2L1*	3	1.21 × 10^−104^	0.598964547956272	0.723	0.338	2.91 × 10^−100^
*TPX2*	3	1.75 × 10^−102^	0.719187603010911	0.772	0.371	4.19 × 10^−98^
*PRR11*	3	1.43 × 10^−96^	0.489548530150862	0.598	0.24	3.42 × 10^−92^
*MKI67*	3	2.35 × 10^−96^	0.596910007884026	0.753	0.312	5.62 × 10^−92^
*DDX39A*	3	3.47 × 10^−96^	0.602213577593247	0.808	0.516	8.30 × 10^−92^
*PIMREG*	3	7.42 × 10^−92^	0.399778725345863	0.433	0.139	1.78 × 10^−87^
*SKA2*	3	1.39 × 10^−89^	0.554764456829379	0.84	0.534	3.33 × 10^−85^
*CKAP2*	3	9.51 × 10^−88^	0.844708042203515	0.731	0.404	2.28 × 10^−84^
*CENPN*	3	7.43 × 10^−87^	0.485581470062575	0.679	0.334	1.78 × 10^−82^
*CENPM*	3	6.78 × 10^−86^	0.496753162630272	0.651	0.303	1.62 × 10^−81^
*TK1*	3	3.41 × 10^−80^	0.489549105877891	0.729	0.36	8.16 × 10^−76^
*SMC4*	3	4.70 × 10^−80^	0.625204041290138	0.867	0.522	1.12 × 10^−75^
*NUSAP1*	3	1.54 × 10^−77^	0.423643896935689	0.799	0.353	3.68 × 10^−73^
*HMMR*	3	9.63 × 10^−77^	0.5858548544598	0.606	0.268	2.31 × 10^−72^
*TACC3*	3	8.69 × 10^−75^	0.488847321071564	0.672	0.31	2.08 × 10^−70^
*ASPM*	3	3.16 × 10^−73^	0.587859571128048	0.635	0.292	7.57 × 10^−68^
*ANP32E*	3	3.27 × 10^−67^	0.520532490104568	0.818	0.561	7.83 × 10^−63^
*KIF20B*	3	7.51 × 10^−66^	0.566391401995441	0.658	0.329	1.80 × 10^−61^
*RPL39L*	3	1.40 × 10^−63^	0.449433426665234	0.729	0.412	3.36 × 10^−59^
*HOTAIRM1*	3	9.20 × 10^−62^	0.539538061574298	0.722	0.486	2.20 × 10^−56^
*NASP*	3	1.66 × 10^−58^	0.481350708319828	0.857	0.615	3.98 × 10^−54^
*EMP2*	3	7.00 × 10^−55^	0.437325491780974	0.703	0.416	1.68 × 10^−50^
*SNHG19*	3	4.24 × 10^−53^	0.356176367815828	0.42	0.184	1.02 × 10^−48^
*MZT1*	3	2.55 × 10^−52^	0.45252067702474	0.672	0.425	6.12 × 10^−48^
*PLK1*	3	5.14 × 10^−52^	0.363157432767263	0.48	0.211	1.23 × 10^−47^
*NEK2*	3	3.87 × 10^−48^	0.303008972860681	0.349	0.134	9.28 × 10^−44^
*KPNA2*	3	4.96 × 10^−45^	0.408224476401982	0.855	0.623	1.19 × 10^−40^
*CENPE*	3	9.27 × 10^−45^	0.429963395668457	0.589	0.303	2.22 × 10^−40^
*NOP58*	3	1.29 × 10^−43^	0.398497263591403	0.7	0.482	3.08 × 10^−40^
*CENPH*	3	5.09 × 10^−43^	0.342977126521325	0.562	0.309	1.22 × 10^−38^
*RUVBL2*	3	4.62 × 10^−43^	0.401938142708255	0.643	0.428	1.11 × 10^−37^
*TYMS*	3	1.75 × 10^−41^	0.348245582386649	0.774	0.442	4.20 × 10^−37^
*NUDT1*	3	1.27 × 10^−40^	0.387242464109805	0.712	0.49	3.03 × 10^−35^
*KNSTRN*	3	4.48 × 10^−39^	0.419295075117713	0.557	0.319	1.07 × 10^−35^
*SMC2*	3	1.60 × 10^−38^	0.363784568686639	0.747	0.508	3.82 × 10^−33^
*SNRPA*	3	4.23 × 10^−36^	0.37927864885733	0.584	0.375	1.01 × 10^−31^
*DNMT1*	3	2.02 × 10^−36^	0.394715713862948	0.785	0.564	4.84 × 10^−31^
*NUF2*	3	8.63 × 10^−30^	0.321669355708339	0.493	0.262	2.07 × 10^−25^
*EZR*	3	6.54 × 10^−28^	0.338204074306678	0.665	0.451	1.57 × 10^−23^
*NDC80*	3	1.26 × 10^−27^	0.319825238579957	0.48	0.26	3.01 × 10^−23^
*SGO2*	3	5.73 × 10^−25^	0.392584539338203	0.545	0.331	1.37 × 10^−20^

**Table 4:** Highly variable genes after cell cycle regression in CAFs isolated from Patient3.

Gene	Cluster	*p*-Value	Average log2FC	Pct.1	Pct.2	*p*-Value Adjusted
*GDF15*	0	1.00 × 10^−124^	0.670012236981009	0.517	0.238	2.33 × 10^−119^
*CEMIP*	0	4.81 × 10^−115^	0.790192919194519	0.621	0.347	1.12 × 10^−110^
*DEPTOR*	0	5.87 × 10^−106^	0.354082730670604	0.379	0.139	1.36 × 10^−101^
*MXD4*	0	6.26 × 10^−90^	0.393580712819188	0.642	0.379	1.45 × 10^−85^
*ARMC9*	0	9.84 × 10^−81^	0.453091970457053	0.676	0.438	2.28 × 10^−77^
*PNRC1*	0	2.58 × 10^−77^	0.31718171845308	0.448	0.216	5.98 × 10^−74^
*CACNB4*	0	2.37 × 10^−74^	0.434880339385922	0.543	0.304	5.50 × 10^−69^
*PLXDC2*	0	7.28 × 10^−68^	0.303863251658921	0.427	0.209	1.69 × 10^−62^
*KIF26B*	0	1.29 × 10^−56^	0.466385578865574	0.589	0.376	2.98 × 10^−52^
*ANKRD37*	0	2.18 × 10^−56^	0.502529523475167	0.482	0.279	5.05 × 10^−52^
*FAM43A*	0	6.47 × 10^−57^	0.341495945768616	0.469	0.257	1.50 × 10^−51^
*TOP2A*	1	0	1.72704801568497	0.862	0.345	0
*UBE2C*	1	0	1.33497306832552	0.733	0.217	0
*HMGB2*	1	0	1.31068678526262	0.911	0.399	0
*HIST1H1B*	1	0	1.21011344182568	0.614	0.102	0
*CDK1*	1	0	1.20899649346328	0.737	0.13	0
*MKI67*	1	0	1.03431981185335	0.81	0.353	0
*DIAPH3*	1	0	1.00041486814025	0.945	0.6	0
*CDCA8*	1	0	0.779702240086265	0.595	0.12	0
*HJURP*	1	0	0.765459493666479	0.573	0.082	0
*CDCA2*	1	0	0.702274359748242	0.565	0.121	0
*GTSE1*	1	0	0.693761336263562	0.604	0.16	0
*NCAPG*	1	0	0.659818505587877	0.627	0.172	0
*AURKB*	1	0	0.619116521095821	0.539	0.108	0
*FBXO5*	1	0	0.615389481482515	0.525	0.103	0
*CKAP2L*	1	0	0.598792006314765	0.541	0.103	0
*ARHGAP11B*	1	0	0.5812417345836	0.473	0.074	0
*KIF18B*	1	0	0.580142879752472	0.471	0.071	0
*KIF23*	1	2.27 × 10^−287^	0.720638959800598	0.691	0.235	5.27 × 10^−283^
*HIST1H1A*	1	6.01 × 10^−285^	0.913609800701636	0.481	0.106	1.39 × 10^−280^
*SMC4*	1	7.58 × 10^−283^	0.864979422051172	0.9	0.514	1.76 × 10^−278^
*ESCO2*	1	2.48 × 10^−281^	0.502365836533897	0.458	0.084	5.75 × 10^−277^
*APOLD1*	1	4.64 × 10^−279^	0.838368640254338	0.67	0.237	1.07 × 10^−274^
*KIF2C*	1	7.56 × 10^−278^	0.589787736752698	0.49	0.104	1.75 × 10^−273^
*MELK*	1	3.52 × 10^−274^	0.575736739467258	0.583	0.16	8.15 × 10^−270^
*TMPO*	1	4.30 × 10^−274^	0.767947092480434	0.843	0.423	9.96 × 10^−270^
*CLSPN*	1	6.95 × 10^−274^	0.719876368800088	0.743	0.275	1.61 × 10^−269^
*TACC3*	1	1.79 × 10^−270^	0.748458380464173	0.717	0.263	4.16 × 10^−266^
*LINC01572*	1	1.38 × 10^−268^	0.589935230882103	0.527	0.134	3.20 × 10^−263^
*NEIL3*	1	4.34 × 10^−266^	0.437098081258912	0.392	0.061	1.01 × 10^−261^
*BRIP1*	1	6.60 × 10^−264^	0.637712916789726	0.588	0.169	1.53 × 10^−259^
*KIF20B*	1	2.62 × 10^−259^	0.726463701130549	0.699	0.258	6.08 × 10^−255^
*CEP128*	1	3.30 × 10^−259^	0.550822882209233	0.495	0.118	7.64 × 10^−255^
*ASPM*	1	5.17 × 10^−252^	0.820869581321595	0.688	0.251	1.20 × 10^−247^
*KNL1*	1	9.01 × 10^−249^	0.595358414126582	0.607	0.184	2.09 × 10^−244^
*KIF11*	1	4.17 × 10^−248^	0.607181845238362	0.591	0.18	9.66 × 10^−244^
*KIFC1*	1	5.13 × 10^−246^	0.485167534071316	0.476	0.108	1.19 × 10^−241^
*NDC80*	1	5.91 × 10^−245^	0.581545868791417	0.579	0.167	1.37 × 10^−240^
*CENPE*	1	1.74 × 10^−242^	0.830486761428012	0.665	0.245	4.03 × 10^−238^
*NUF2*	1	6.57 × 10^−242^	0.638010395689706	0.612	0.197	1.52 × 10^−238^
*CENPU*	1	4.52 × 10^−240^	0.581598609771616	0.698	0.252	1.05 × 10^−235^
*TTK*	1	7.67 × 10^−238^	0.437592463155119	0.405	0.076	1.78 × 10^−233^
*MIR924HG*	1	1.21 × 10^−238^	0.647701379585797	0.516	0.138	2.81 × 10^−233^
*DBF4*	1	7.15 × 10^−237^	0.632464785381952	0.653	0.242	1.66 × 10^−232^
*CENPK*	1	1.91 × 10^−233^	0.62044231564181	0.718	0.292	4.42 × 10^−229^
*SGO2*	1	2.05 × 10^−232^	0.667120708929706	0.645	0.229	4.75 × 10^−228^
*CCNA2*	1	1.22 × 10^−231^	0.623778974117052	0.631	0.216	2.84 × 10^−228^
*ATAD2*	1	3.59 × 10^−229^	0.658767565339001	0.721	0.297	8.32 × 10^−225^
*ORC6*	1	2.23 × 10^−227^	0.481784925937081	0.52	0.141	5.18 × 10^−223^
*SPC25*	1	1.19 × 10^−225^	0.581833584432011	0.592	0.191	2.75 × 10^−221^
*KIF14*	1	2.21 × 10^−224^	0.490496690709161	0.459	0.109	5.11 × 10^−220^
*UBE2T*	1	4.57 × 10^−224^	0.621313085775268	0.733	0.313	1.06 × 10^−219^
*HIST1H1E*	1	3.81 × 10^−217^	0.722266888541442	0.527	0.175	8.82 × 10^−213^
*KIF4A*	1	6.36 × 10^−216^	0.543875954867782	0.56	0.175	1.47 × 10^−212^
*GMNN*	1	1.53 × 10^−215^	0.583998264523595	0.736	0.317	3.54 × 10^−211^
*FEN1*	1	2.71 × 10^−213^	0.507380219199132	0.583	0.19	6.29 × 10^−209^
*NUSAP1*	1	2.89 × 10^−212^	0.772936396606703	0.763	0.371	6.70 × 10^−208^
*CKS2*	1	3.74 × 10^−212^	0.871013874667081	0.883	0.609	8.66 × 10^−208^
*NSD2*	1	1.62 × 10^−211^	0.630784667555947	0.748	0.36	3.77 × 10^−207^
*AURKA*	1	5.76 × 10^−210^	0.734078391529851	0.582	0.203	1.34 × 10^−205^
*CENPF*	1	4.08 × 10^−212^	0.981493266552953	0.836	0.46	9.46 × 10^−205^
*ANLN*	1	5.04 × 10^−208^	0.665580161160135	0.71	0.305	1.17 × 10^−203^
*CCDC18*	1	2.17 × 10^−205^	0.537992631230034	0.589	0.209	5.03 × 10^−201^
*SGO1*	1	2.14 × 10^−204^	0.42462140086729	0.445	0.109	4.95 × 10^−200^
*BARD1*	1	1.55 × 10^−204^	0.469675415789094	0.51	0.152	3.60 × 10^−199^
*DLEU2*	1	9.72 × 10^−202^	0.600325914395249	0.681	0.297	2.25 × 10^−197^
*HIST1H3B*	1	8.08 × 10^−198^	0.331364514860151	0.281	0.037	1.87 × 10^−193^
*KIF15*	1	3.79 × 10^−197^	0.366987727162554	0.367	0.074	8.79 × 10^−193^
*TPX2*	1	3.06 × 10^−193^	0.732127237168298	0.831	0.458	7.09 × 10^−189^
*KIF18A*	1	3.14 × 10^−192^	0.510434190240487	0.535	0.178	7.28 × 10^−188^
*SPDL1*	1	5.37 × 10^−191^	0.639004145224719	0.805	0.474	1.24 × 10^−186^
*PBK*	1	6.00 × 10^−188^	0.652609738045231	0.774	0.375	1.39 × 10^−183^
*BUB1B*	1	6.41 × 10^−186^	0.447366477932345	0.494	0.146	1.49 × 10^−181^
*RFC3*	1	6.72 × 10^−185^	0.442017047728623	0.501	0.154	1.56 × 10^−180^
*ARHGAP11A*	1	2.57 × 10^−184^	0.44833851287311	0.427	0.114	5.96 × 10^−180^
*CDC25C*	1	8.49 × 10^−184^	0.302666270199418	0.274	0.038	1.97 × 10^−179^
*USP1*	1	9.57 × 10^−184^	0.554036641164941	0.716	0.339	2.22 × 10^−179^
*H2AFX*	1	3.44 × 10^−183^	0.620529441343685	0.712	0.359	7.98 × 10^−179^
*CDCA4*	1	1.80 × 10^−183^	0.421167860141002	0.469	0.141	4.16 × 10^−178^
*RRM2*	1	7.34 × 10^−182^	0.5714047398839	0.659	0.276	1.70 × 10^−177^
*POLQ*	1	1.14 × 10^−181^	0.334881354743659	0.338	0.067	2.65 × 10^−176^
*BRCA1*	1	1.27 × 10^−179^	0.463882543405512	0.533	0.18	2.94 × 10^−176^
*C21orf58*	1	3.34 × 10^−174^	0.349629662415553	0.366	0.084	7.74 × 10^−170^
*CENPA*	1	3.60 × 10^−173^	0.501865111481393	0.454	0.139	8.36 × 10^−170^
*CIT*	1	7.99 × 10^−173^	0.414358233464705	0.423	0.117	1.85 × 10^−168^
*RAD51AP1*	1	2.60 × 10^−172^	0.393885717817882	0.417	0.111	6.02 × 10^−168^
*KPNA2*	1	8.94 × 10^−171^	0.852269471899063	0.858	0.593	2.07 × 10^−166^
*GAS2L3*	1	2.47 × 10^−169^	0.425123516789511	0.376	0.094	5.72 × 10^−165^
*FAM83D*	1	1.28 × 10^−166^	0.355532271296998	0.325	0.068	2.96 × 10^−162^
*DNAJC9*	1	9.66 × 10^−166^	0.539087426376657	0.8	0.458	2.24 × 10^−161^
*MIS18BP1*	1	2.18 × 10^−163^	0.526547560532024	0.672	0.321	5.06 × 10^−159^
*TYMS*	1	6.28 × 10^−163^	0.567080173939906	0.846	0.508	1.46 × 10^−158^
*CKAP2*	1	3.26 × 10^−162^	0.695663722723763	0.709	0.361	7.56 × 10^−158^
*NCAPH*	1	8.18 × 10^−162^	0.310271769913698	0.338	0.074	1.90 × 10^−157^
*BRCA2*	1	1.09 × 10^−161^	0.413729693225856	0.444	0.136	2.53 × 10^−157^
*FAM111A*	1	2.02 × 10^−161^	0.399559533411284	0.439	0.135	4.69 × 10^−158^
*ECT2*	1	1.61 × 10^−160^	0.477856374138588	0.562	0.218	3.74 × 10^−156^
*HMMR*	1	1.47 × 10^−150^	0.551172165466593	0.637	0.275	3.41 × 10^−146^
*MAD2L1*	1	1.96 × 10^−149^	0.451799259093044	0.638	0.272	4.55 × 10^−145^
*SHCBP1*	1	3.08 × 10^−147^	0.423856719181729	0.536	0.201	7.13 × 10^−144^
*HIST1H1C*	1	1.37 × 10^−145^	0.608927235709592	0.559	0.255	3.18 × 10^−141^
*MXD3*	1	7.58 × 10^−143^	0.38986107239568	0.387	0.114	1.76 × 10^−138^
*DNMT1*	1	1.44 × 10^−142^	0.515550569349073	0.9	0.66	3.34 × 10^−138^
*NAV2*	1	3.85 × 10^−142^	0.688091984444401	0.776	0.512	8.92 × 10^−138^
*CDC45*	1	6.33 × 10^−141^	0.310435808801802	0.366	0.098	1.47 × 10^−136^
*PIF1*	1	5.35 × 10^−139^	0.36016834161781	0.292	0.067	1.24 × 10^−134^
*MYBL1*	1	1.80 × 10^−136^	0.318318334241842	0.347	0.093	4.17 × 10^−132^
*VRK1*	1	5.73 × 10^−136^	0.392139040630886	0.502	0.19	1.33 × 10^−131^
*ZWINT*	1	6.04 × 10^−137^	0.451302935397852	0.61	0.277	1.40 × 10^−131^
*DTL*	1	3.02 × 10^−135^	0.367072327396311	0.372	0.108	7.00 × 10^−131^
*SMC2*	1	4.91 × 10^−133^	0.49254004897609	0.802	0.489	1.14 × 10^−128^
*HELLS*	1	4.44 × 10^−131^	0.472491197382194	0.597	0.269	1.03 × 10^−126^
*SCLT1*	1	5.28 × 10^−131^	0.550006155368793	0.677	0.377	1.22 × 10^−126^
*CEP55*	1	1.14 × 10^−130^	0.490879947494692	0.684	0.333	2.64 × 10^−126^
*MND1*	1	8.76 × 10^−130^	0.312301643925559	0.361	0.103	2.03 × 10^−125^
*SKA3*	1	4.05 × 10^−127^	0.310432816536786	0.354	0.102	9.39 × 10^−123^
*MCM7*	1	3.48 × 10^−125^	0.43694530973709	0.662	0.317	8.07 × 10^−121^
*FOSL1*	1	1.48 × 10^−125^	0.579087551857356	0.838	0.614	3.44 × 10^−120^
*RAD18*	1	6.07 × 10^−124^	0.369091089371499	0.493	0.194	1.41 × 10^−119^
*EZH2*	1	2.68 × 10^−122^	0.350087093415413	0.43	0.151	6.21 × 10^−118^
*SPATA5*	1	4.54 × 10^−121^	0.430333880167729	0.539	0.245	1.05 × 10^−116^
*MIS18A*	1	5.92 × 10^−120^	0.338627347295294	0.455	0.167	1.37 × 10^−116^
*NCAPG2*	1	8.08 × 10^−120^	0.388953597980289	0.518	0.212	1.87 × 10^−115^
*HIST2H2AC*	1	2.18 × 10^−117^	0.459827084117736	0.447	0.181	5.04 × 10^−113^
*CIP2A*	1	2.48 × 10^−118^	0.348589258463476	0.443	0.164	5.75 × 10^−113^
*NCAPD3*	1	4.68 × 10^−118^	0.364429029867863	0.498	0.205	1.08 × 10^−112^
*PCLAF*	1	3.77 × 10^−116^	0.458137696175664	0.862	0.584	8.74 × 10^−112^
*CENPP*	1	6.27 × 10^−116^	0.415419981923284	0.506	0.212	1.45 × 10^−111^
*DEPDC1*	1	1.61 × 10^−113^	0.393812693913351	0.516	0.211	3.72 × 10^−109^
*PRIM2*	1	4.97 × 10^−113^	0.422873887917786	0.692	0.372	1.15 × 10^−112^
*RRM1*	1	8.12 × 10^−113^	0.431960647746961	0.665	0.355	1.88 × 10^−108^
*G2E3*	1	1.51 × 10^−112^	0.444785977826341	0.585	0.296	3.50 × 10^−108^
*PCNA*	1	3.48 × 10^−112^	0.476948583142726	0.661	0.369	8.06 × 10^−108^
*AP001347.1*	1	1.12 × 10^−111^	0.315812948836076	0.373	0.125	2.60 × 10^−107^
*CKS1B*	1	3.12 × 10^−109^	0.456582258564046	0.778	0.479	7.24 × 10^−105^
*GPSM2*	1	1.17 × 10^−108^	0.345728943259348	0.417	0.156	2.72 × 10^−105^
*PRC1*	1	2.53 × 10^−107^	0.476953071781039	0.783	0.449	5.87 × 10^−103^
*KNSTRN*	1	9.23 × 10^−107^	0.401461259105815	0.512	0.225	2.14 × 10^−102^
*RACGAP1*	1	1.35 × 10^−106^	0.332736847877824	0.411	0.15	3.13 × 10^−103^
*PLK1*	1	2.35 × 10^−106^	0.412673618186031	0.46	0.184	5.44 × 10^−102^
*FANCI*	1	3.69 × 10^−107^	0.324134569142021	0.433	0.164	8.55 × 10^−102^
*ITGB3BP*	1	9.35 × 10^−105^	0.36277717625636	0.596	0.287	2.17 × 10^−100^
*POLD3*	1	1.37 × 10^−103^	0.348618544428778	0.461	0.189	3.17 × 10^−99^
*DTYMK*	1	2.55 × 10^−103^	0.432705404552704	0.902	0.692	5.91 × 10^−99^
*TRIM59*	1	2.03 × 10^−98^	0.38570544217349	0.509	0.237	4.70 × 10^−94^
*RFC4*	1	1.74 × 10^−97^	0.353796335374352	0.489	0.215	4.03 × 10^−93^
*CHAF1A*	1	8.15 × 10^−97^	0.310489196440483	0.409	0.156	1.89 × 10^−92^
*MCM5*	1	1.35 × 10^−96^	0.34553837929917	0.536	0.241	3.14 × 10^−92^
*GINS2*	1	7.46 × 10^−95^	0.34719563516437	0.518	0.233	1.73 × 10^−91^
*CDKN2D*	1	2.50 × 10^−94^	0.368325034441899	0.391	0.155	5.79 × 10^−90^
*PGP*	1	1.02 × 10^−93^	0.407835180695608	0.798	0.544	2.37 × 10^−89^
*SMC3*	1	4.64 × 10^−91^	0.408844500543907	0.836	0.62	1.08 × 10^−86^
*NASP*	1	5.28 × 10^−84^	0.395402376975593	0.847	0.611	1.22 × 10^−80^
*CKAP5*	1	1.46 × 10^−83^	0.410491352853466	0.67	0.419	3.39 × 10^−78^
*CBX5*	1	2.20 × 10^−82^	0.369518182859071	0.864	0.652	5.10 × 10^−78^
*CCDC14*	1	2.74 × 10^−81^	0.308855146567134	0.455	0.207	6.35 × 10^−77^
*SMC1A*	1	1.81 × 10^−78^	0.376337526793547	0.655	0.389	4.20 × 10^−74^
*CENPM*	1	1.94 × 10^−77^	0.329745149819119	0.597	0.316	4.50 × 10^−73^
*BUB3*	1	3.08 × 10^−76^	0.401293068494684	0.797	0.586	7.15 × 10^−73^
*PRR11*	1	9.66 × 10^−76^	0.356696418545198	0.542	0.28	2.24 × 10^−71^
*SAP30*	1	1.49 × 10^−75^	0.340983727579872	0.555	0.296	3.45 × 10^−71^
*SAC3D1*	1	2.96 × 10^−74^	0.345588725644688	0.588	0.333	6.87 × 10^−71^
*DHFR*	1	1.98 × 10^−72^	0.309844463962329	0.513	0.256	4.60 × 10^−68^
*EMP2*	1	5.47 × 10^−72^	0.308138970460742	0.537	0.275	1.27 × 10^−67^
*CMC2*	1	2.34 × 10^−70^	0.341531617753068	0.725	0.479	5.42 × 10^−66^
*POLE3*	1	1.23 × 10^−67^	0.306632137012119	0.586	0.327	2.85 × 10^−64^
*BIRC5*	1	4.30 × 10^−66^	0.364636043735149	0.827	0.572	9.97 × 10^−62^
*PSIP1*	1	4.97 × 10^−66^	0.335570400412872	0.814	0.591	1.15 × 10^−61^
*CDK5RAP2*	1	2.04 × 10^−61^	0.303897510950978	0.533	0.293	4.74 × 10^−57^
*PARP1*	1	4.80 × 10^−61^	0.337561777573747	0.749	0.529	1.11 × 10^−56^
*LCORL*	1	5.83 × 10^−61^	0.329713081181335	0.489	0.268	1.35 × 10^−56^
*BTG3*	1	1.85 × 10^−60^	0.332337133755457	0.717	0.477	4.28 × 10^−56^
*TK1*	1	2.04 × 10^−59^	0.33510181879942	0.807	0.59	4.72 × 10^−55^
*RAD51B*	1	2.52 × 10^−58^	0.331080583017355	0.566	0.335	5.84 × 10^−54^
*CENPW*	1	2.58 × 10^−54^	0.308998607277912	0.863	0.629	5.97 × 10^−50^
*CDC20*	1	5.43 × 10^−52^	0.318295484247153	0.642	0.382	1.26 × 10^−47^
*EXOSC9*	1	6.81 × 10^−54^	0.304012196032269	0.585	0.364	1.58 × 10^−46^
*KMT5A*	1	2.58 × 10^−50^	0.306185177005478	0.597	0.378	5.98 × 10^−46^
*RAD21*	1	3.48 × 10^−50^	0.319947957948111	0.715	0.503	8.07 × 10^−46^
*NCEH1*	1	7.36 × 10^−49^	0.310793306903988	0.558	0.344	1.71 × 10^−44^
*CCNB1*	2	0	1.31153913591001	0.924	0.423	0
*CDC20*	2	0	1.12129311051783	0.867	0.348	0
*CENPF*	2	0	1.04026677851946	0.956	0.461	0
*PTTG1*	2	0	1.03054711379723	0.984	0.7	0
*DLGAP5*	2	0	0.917754645754222	0.753	0.268	0
*CCNB2*	2	1.58 × 10^−249^	0.825103171657037	0.726	0.301	3.67 × 10^−246^
*PRC1*	2	3.33 × 10^−244^	0.871240943557588	0.866	0.455	7.71 × 10^−239^
*TPX2*	2	4.54 × 10^−235^	0.876780210888109	0.888	0.474	1.05 × 10^−231^
*CDKN3*	2	5.29 × 10^−233^	0.865324099602013	0.902	0.553	1.23 × 10^−228^
*BIRC5*	2	2.76 × 10^−211^	0.795052317508702	0.908	0.572	6.40 × 10^−206^
*PBK*	2	1.49 × 10^−204^	0.787661110394609	0.819	0.395	3.45 × 10^−200^
*ARL6IP1*	2	6.80 × 10^−195^	0.816866773874749	0.814	0.494	1.58 × 10^−190^
*HMMR*	2	1.85 × 10^−183^	0.715432914553575	0.685	0.292	4.29 × 10^−179^
*CEP55*	2	1.66 × 10^−175^	0.704510401183117	0.727	0.35	3.86 × 10^−171^
*MKI67*	2	2.89 × 10^−160^	0.644584202642253	0.812	0.388	6.70 × 10^−156^
*NUSAP1*	2	2.03 × 10^−150^	0.648434446726603	0.778	0.398	4.70 × 10^−146^
*CDCA3*	2	1.88 × 10^−138^	0.589370038211954	0.568	0.252	4.36 × 10^−134^
*SMC4*	2	2.00 × 10^−131^	0.608459878088531	0.869	0.551	4.64 × 10^−128^
*CKAP2*	2	4.48 × 10^−117^	0.680097827457623	0.694	0.392	1.04 × 10^−112^
*CKS1B*	2	2.31 × 10^−108^	0.559775788818762	0.77	0.504	5.35 × 10^−104^
*PHF19*	2	2.70 × 10^−103^	0.53926975121795	0.701	0.455	6.27 × 10^−98^
*DEPDC1*	2	2.72 × 10^−99^	0.517613684298651	0.505	0.238	6.30 × 10^−95^
*HMGB3*	2	7.45 × 10^−90^	0.522062416887759	0.722	0.513	1.73 × 10^−86^
*KPNA2*	2	1.33 × 10^−88^	0.442058824158783	0.831	0.62	3.07 × 10^−83^
*SNHG3*	2	4.26 × 10^−85^	0.515930886429753	0.55	0.339	9.87 × 10^−81^
*ANLN*	2	1.74 × 10^−77^	0.455210659452447	0.625	0.357	4.04 × 10^−72^
*PLK1*	2	9.38 × 10^−72^	0.422891321882443	0.444	0.21	2.17 × 10^−67^
*MAD2L1*	2	2.06 × 10^−71^	0.468184987478444	0.55	0.322	4.78 × 10^−67^
*TACC3*	2	9.49 × 10^−70^	0.425832817667313	0.59	0.329	2.20 × 10^−64^
*CENPE*	2	8.03 × 10^−67^	0.433653107799618	0.563	0.302	1.86 × 10^−62^
*ASPM*	2	2.51 × 10^−62^	0.465087211115013	0.558	0.317	5.83 × 10^−58^
*KIF20B*	2	7.25 × 10^−44^	0.359035439133911	0.542	0.33	1.68 × 10^−40^
*PTTG1*	3	1.81 × 10^−58^	0.459413851506037	0.93	0.724	4.19 × 10^−54^

**Table 5:** Shared highly variable genes identified from CAFs/DAFs isolated from chronic pancreatitis and pancreatic cancer patients.

*ANLN, APOLD1, ARHGAP11A, ARH11B, ASPM, ATAD2, AURKA, AURKB, BARD1, BIRC5, BRCA1, BRCA2, BRIP1, BUB1B, C21orf58, CCDC14, CCDC18, CCNA2, CCNB1, CCNB2, CDC20, CDC45, CDCA2, CDCA3, CDCA4, CDCA8, CDKN2D, CDKN3, CENPA, CENPE, CENPF, CENPK, CENPM, CENPP, CENPU, CEP128, CEP55, CIP2A, CIT, CKAP2, CKAP2L, CLSPN, DBF4, DEPDC1, DHFR, DLEU2, DLGAP5, DNAJC9, ECT2, EMP2, ESCO2, EZH2, FAM111A, FANCI, FBXO5, FEN1, G2E3, GAS2L3, GINS2, GMNN, GPSM2, GTSE1, H2AFX, HELLS, HIST1H1A, HIST1H1B, HIST1H1E, HJURP, HMGB2, HMMR, KIF11, KIF14, KIF18A, KIF18B, KIF20B, KIF23, KIF2C, KIF4A, KIFC1, KNL1, KNSTRN, LINC01572, MAD2L1, MCM7, MELK, MIR924HG, MIS18BP1, MKI67, MND1, MXD3, MYBL1, NAV2, NCAPG, NCAPG2, NCAPH, NDC80, NEIL3, NSD2, NUF2, NUSAP1, ORC6, PBK, PCLAF, PHF19, PLK1, POLD3, PRC1, PRIM2, PRR11, RACGAP1, RAD51AP1, RFC3, RRM1, RRM2, SACD1, SAP30, SCLT1, SGO1, SGO2, SHCBP1, SMC1A, SMC2, SMC4, SPC25, TACC3, TMPO, TOP2A, TPX2, TRIM59, TTK, UBE2C, UBE2T, USP1, VRK1, ZWINT*

**Table 6:** Lists of selected markers for myCAF, iCAF, and apCAF.

CAF Subtype	Selected Markers
myCAF (Öhlund et al.) [10]	*Acta2, Vim, Ctgf, Col1a1, Col5a1, Col6a1*
myCAF (Elyada et al.) [11]	*Tnc, Tgfb1, Thy1, Tagln, Col12a1, Pdgfrb*
iCAF (Öhlund et al.) [10]	*Il1, Il6, Il11, Lif*
iCAF (Elyada et al.) [11]	*Clec3b, Col14a1, Gsn, Ly6c1, Cxcl12*
apCAF (Elyada et al.) [11]	*Slpi, Saa3, Cd74, H2-Ab1, NKain4, Irf5*

## Data Availability

Sequencing raw data are available at European Genome-Phenome Archive (EGA) box with the accession number EGAS00001005889.

## References

[1] Siegel R.L., Miller K.D., Fuchs H.E., Jemal A. (2022). Cancer statistics. CA Cancer J. Clin..

[2] Rahib L., Smith B.D., Aizenberg R., Rosenzweig A.B., Fleshman J.M., Matrisian L.M. (2014). Projecting cancer incidence and deaths to 2030: The unexpected burden of thyroid, liver, and pancreas cancers in the United States. Cancer Res..

[3] Kleeff J., Korc M., Apte M., La Vecchia C., Johnson C.D., Biankin A.V., Neale R.E., Tempero M., Tuveson D.A., Hruban R.H. (2016). Pancreatic cancer. Nat. Rev. Dis. Primers.

[4] Sunami Y., Häußler J., Kleeff J. (2020). Cellular Heterogeneity of Pancreatic Stellate Cells, Mesenchymal Stem Cells, and Cancer-Associated Fibroblasts in Pancreatic Cancer. Cancers.

[5] Sunami Y., Böker V., Kleeff J. (2021). Targeting and Reprograming Cancer-Associated Fibroblasts and the Tumor Microenvironment in Pancreatic Cancer. Cancers.

[6] Murphy K.J., Chambers C.R., Herrmann D., Timpson P., Pereira B.A. (2021). Dynamic Stromal Alterations Influence Tumor-Stroma Crosstalk to Promote Pancreatic Cancer and Treatment Resistance. Cancers.

[7] Barrera L.N., Evans A., Lane B., Brumskill S., Oldfield F.E., Campbell F., Andrews T., Lu Z., Perez-Mancera P.A., Liloglou T. (2020). Fibroblasts from Distinct Pancreatic Pathologies Exhibit Disease-Specific Properties. Cancer Res..

[8] Erkan M., Adler G., Apte M.V., Bachem M.G., Buchholz M., Detlefsen S., Esposito I., Friess H., Gress T.M., Habisch H.J. (2012). StellaTUM: Current consensus and discussion on pancreatic stellate cell research. Gut.

[9] Biffi G., Tuveson D.A. (2021). Diversity and Biology of Cancer-Associated Fibroblasts. Physiol. Rev..

[10] Öhlund D., Handly-Santana A., Biffi G., Elyada E., Almeida A.S., Ponz-Sarvise M., Corbo V., Oni T.E., Hearn S.A., Lee E.J. (2017). Distinct populations of inflammatory fibroblasts and myofibroblasts in pancreatic cancer. J. Exp. Med..

[11] Elyada E., Bolisetty M., Laise P., Flynn W.F., Courtois E.T., Burkhart R.A., Teinor J.A., Belleau P., Biffi G., Lucito M.S. (2019). Cross-Species Single-Cell Analysis of Pancreatic Ductal Adenocarcinoma Reveals Antigen-Presenting Cancer-Associated Fibroblasts. Cancer Discov..

[12] Bachem M.G., Schneider E., Gross H., Weidenbach H., Schmid R.M., Menke A., Siech M., Beger H., Grünert A., Adler G. (1998). Identification, culture, and characterization of pancreatic stellate cells in rats and humans. Gastroenterology.

[13] Tosti L., Hang Y., Debnath O., Tiesmeyer S., Trefzer T., Steiger K., Ten F.W., Lukassen S., Ballke S., Kühl A.A. (2021). Single-Nucleus and In Situ RNA-Sequencing Reveal Cell Topographies in the Human Pancreas. Gastroenterology.

[14] Tang Z., Li C., Kang B., Gao G., Li C., Zhang Z. (2017). GEPIA: A web server for cancer and normal gene expression profiling and interactive analyses. Nucleic Acids Res..

[15] Bai R., Rebelo A., Kleeff J., Sunami Y. (2021). Identification of prognostic lipid droplet-associated genes in pancreatic cancer patients via bioinformatics analysis. Lipids Health Dis..

[16] Helms E.J., Berry M.W., Chaw R.C., DuFort C.C., Sun D., Onate M.K., Oon C., Bhattacharyya S., Sanford-Crane H., Horton W. (2022). Mesenchymal Lineage Heterogeneity Underlies Nonredundant Functions of Pancreatic Cancer-Associated Fibroblasts. Cancer Discov..

[17] Tirosh I., Izar B., Prakadan S.M., Wadsworth MH 2nd Treacy D., Trombetta J.J., Rotem A., Rodman C., Lian C., Murphy G., Fallahi-Sichani M. (2016). Dissecting the multicellular ecosystem of metastatic melanoma by single-cell RNA-seq. Science.

[18] Huang S., Zhao J., Song J., Li Y., Zuo R., Sa Y., Ma Z., OuYang H. (2021). Interferon alpha-inducible protein 27 (IFI27) is a prognostic marker for pancreatic cancer based on comprehensive bioinformatics analysis. Bioengineered.

[19] Lao M., Zhang X., Ma T., Xu J., Yang H., Duan Y., Ying H., Zhang X., Guo C., Qiu J. (2021). Regulator of calcineurin 1 gene isoform 4 in pancreatic ductal adenocarcinoma regulates the progression of tumor cells. Oncogene.

[20] Xiong Y., Ju L., Yuan L., Chen L., Wang G., Xu H., Peng T., Luo Y., Xiao Y., Wang X. (2021). KNSTRN promotes tumorigenesis and gemcitabine resistance by activating AKT in bladder cancer. Oncogene.

[21] Chen P., He Z., Wang J., Xu J., Jiang X., Chen Y., Liu X., Jiang J. (2021). Hypoxia-Induced ZWINT Mediates Pancreatic Cancer Proliferation by Interacting With p53/p21. Front. Cell Dev. Biol..

[22] Grünwald B.T., Devisme A., Andrieux G., Vyas F., Aliar K., McCloskey C.W., Macklin A., Jang G.H., Denroche R., Romero J.M. (2021). Spatially confined sub-tumor microenvironments in pancreatic cancer. Cell.

[23] Dominguez C.X., Müller S., Keerthivasan S., Koeppen H., Hung J., Gierke S., Breart B., Foreman O., Bainbridge T.W., Castiglioni A. (2020). Single-Cell RNA Sequencing Reveals Stromal Evolution into LRRC15^+^ Myofibroblasts as a Determinant of Patient Response to Cancer Immunotherapy. Cancer Discov..

